# Fractional exhaled nitric oxide at multiple flow rates in chronic cough: association with lung function and airway hyperresponsiveness

**DOI:** 10.1080/07853890.2026.2681282

**Published:** 2026-06-26

**Authors:** Dan Wang, Yan Zhang, Yun Liu

**Affiliations:** Department of Respiratory and Critical Care Medicine, The Second Affiliated Hospital of Xi’an Jiaotong University, Xi’an, China

**Keywords:** Airway hyperresponsiveness (AHR), chronic cough, fractional exhaled nitric oxide (FeNO), lung function tests

## Abstract

**Background:**

The relationship between airway inflammation and airflow obstruction in patients with chronic cough remains unclear. This study aimed to evaluate the relationships between multi-flow fractional exhaled nitric oxide parameters (FeNO_50_, FeNO_200_, and CaNO) and lung function, and to assess their utility in predicting airway hyperresponsiveness (AHR) in this population.

**Materials and methods:**

This retrospective study enrolled 2205 chronic cough outpatients who underwent spirometry and multiple-flow FeNO measurements. Patients were stratified by airway function and FeNO levels for group comparisons, and Spearman’s rank correlation was used. In a subgroup (*n* = 582) who underwent methacholine bronchial provocation test (BPT), the diagnostic accuracy of these parameters for AHR was evaluated.

**Results:**

Among 2014 patients with normal ventilation or airflow obstruction, FeNO_50_ and FeNO_200_ were significantly higher in outpatients with airflow obstruction than those with both normal ventilation and normal small airway function (*p* < 0.01). Weak but significant negative correlations were found between FeNO_50_ or FeNO_200_ and lung function parameters (all coefficients ∼0.1), which were slightly stronger in females. FeNO_50_ and FeNO_200_ showed moderate diagnostic value (AUCs: 0.775 and 0.721, Cut-off: 31.5 and 14.5 ppb, respectively), while CaNO showed none. Combining FeNO_50_ with small airway parameter (FEF_50_%pred) yielded the highest predictive accuracy (AUC: 0.895).

**Conclusions:**

FeNO_50_ and FeNO_200_ are associated with the severity of airflow obstruction and moderately predict AHR in chronic cough, whereas CaNO does not. Combining FeNO with lung function parameters enhances predictive performance. Despite statistical significance, the weak correlations between FeNO and lung function parameters warrant cautious clinical interpretation.

## Key Messages

1. Elevated FeNO_50_ and FeNO_200_ levels are associated with more severe airflow obstruction and show moderate predictive value for airway hyperresponsiveness (AHR) in patients with chronic cough.
2. Combining FeNO_50_ with the small airway function parameter FEF_50_ (%pred) significantly improves the prediction accuracy for AHR compared with using either marker alone.
3. In contrast to FeNO_50_ and FeNO_200_, alveolar nitric oxide (CaNO) shows minimal association with lung function and no diagnostic utility for AHR in this chronic cough cohort.

## 1. Introduction

Chronic cough, defined as a symptom persisting for more than eight weeks in adults, is a prevalent and diagnostically challenging condition in respiratory medicine [1]. Its etiology is multifactorial, with common causes including cough-variant asthma (CVA), upper airway cough syndrome (UACS), gastroesophageal reflux disease (GERD), and chronic obstructive pulmonary disease (COPD) [2]. A systematic diagnostic evaluation is therefore essential, encompassing assessments of both airway inflammation and airway function—specifically through tests for airflow obstruction or airway hyperresponsiveness (AHR).

Airway inflammation is an important pathogenic driver in chronic cough. Fractional exhaled nitric oxide (FeNO) has emerged as a valuable, non-invasive biomarker for identifying type 2 (T2) airway inflammation. However, its interpretation is complex, being influenced not only by the degree of inflammation but also by airway function and other factors. Notably, airflow obstruction and AHR, measured by spirometry and bronchoprovocation tests (BPT) respectively, are not universal in chronic cough but are critical for distinguishing among COPD, classic asthma and CVA [3].

The interaction between FeNO and airway function is bidirectional and clinically significant. FeNO levels are reduced by narrowing airway caliber, indicating that airflow obstruction itself modulates this inflammatory readout [4]. Meanwhile, persistently elevated FeNO has been associated with accelerated lung function decline in chronic airway diseases [5–8]. In COPD, patients with persistently low FeNO levels had poorer lung function and reported more dyspnea than those with high levels [9]. Furthermore, FeNO at 200 mL/s (FeNO_200_) and alveolar nitric oxide concentration (CaNO), which reflect T2 inflammation in small airways, are elevated in COPD, with negative correlations observed between CaNO and lung function [10,11]. Despite these insights, the specific relationships between multiple-flow FeNO parameters (FeNO_50_, FeNO_200_, and CaNO) and detailed lung function parameters encompassing both large and small airways remain poorly defined in the large real-world population of outpatients with chronic cough.

Additionally, airway hyperresponsiveness (AHR) is a treatable feature of CVA, detected by BPT. While various studies have demonstrated that FeNO_50_ could be a diagnostic indicator for AHR, either alone or combined with lung function parameters [12–16]. However, the utility of a multi-flow FeNO approach for predicting AHR remains largely unexplored. It is unclear whether FeNO_200_ or CaNO, particularly when integrated with comprehensive assessments of both large and small airway function, can enhance diagnostic accuracy.

Given these complex interactions and the lack of data in general chronic cough populations, the present study was designed with the following objectives in a large real-world cohort: 1) To characterize the mutual relationship between multiple-flow FeNO parameters (FeNO_50_, FeNO_200_, and CaNO) and lung function parameters reflecting both large and small airways. 2) To evaluate the diagnostic performance of these FeNO parameters—individually and in combination with lung function parameters—for predicting AHR, and to explore their potential diagnostic cutoff values.

## 2. Methods

### 2.1. Study design and participant selection

We retrospectively collected data from 2205 outpatients at the respiratory clinic of the Second Affiliated Hospital of Xi’an Jiaotong University, China between January 2021 and May 2022. Ethical approval was granted by the Ethics Committee of the Second Affiliated Hospital of Xi’an Jiaotong University (Approval No. 183).

The patients included in this study had a persistent history of chronic cough for 8 weeks, with or without symptoms of expectoration, chest tightness, or wheezing. All patients underwent both spirometry test and multiple flow rates of fractional exhaled nitric oxide (FeNO) measurements (FeNO_50_, FeNO_200_, and CaNO) based on physicians’ opinions.

### 2.2. Data collection

FeNO measurements were performed prior to spirometry and bronchoprovocation tests, following American Thoracic Society (ATS)/European Respiratory Society (ERS) recommendations [17]. Measurements were obtained using a Nano Coulomb breath analyzer (SUNVOU-CA2122, Shangwo Biotech Co., Ltd, Wuxi, China), which employs an auto-adjusted flow-rate sampling technology. Patients were instructed to inhale NO-free air and exhale *via* a mouthpiece at two constant flow rates: 50 mL/s for ≥4 s and 200 mL/s for ≥2 s. The alveolar nitric oxide concentration (CaNO) was estimated using a simplified linear model: CaNO = (FeNO_200_ – FeNO_50_ + Corr)/150, where Corr is a correction coefficient based on interpolation and fitting of extensive literature data and internal experimental datasets [18–21].

Demographic data (sex, height, weight, and age) were extracted from spirometry reports. Spirometry and bronchoprovocation tests (BPT) were performed using a spirometer (Jaeger, Hoechberg, Germany) according to the specifications and performance criteria recommended in the ATS/ERS Standardization of Spirometry [22]. The BPT was performed with the Jaeger APS Pro system using a Medic-Aid sidestream nebulizer and double doses of methacholine (0.078–1.251 mg). The following predicted lung function parameters were recorded for analysis: vital capacity (VC), forced vital capacity (FVC), forced expiratory volume in the first second (FEV_1_), FEV_1_/FVC, FEV_1_/VC, forced expiratory flow at 50% of FVC (FEF_50_), FEF_75_, forced expiratory flow between 25% and 75% (FEF_25–75_), and total lung capacity (TLC).

### 2.3. Grouping methods

Patients were classified into airway function subtypes based on the algorithm outlined in Figure S1 in the supporting information. Based on the predicted values of lung function parameters, patients were grouped into the following subgroups: 1) normal ventilation without small airway dysfunction (N + n-SAD); 2) normal ventilation with small airway dysfunction (N + SAD); 3) mild airflow obstruction; 4) moderate to extreme airflow obstruction; 5) restrictive ventilatory dysfunction; and 6) mixed ventilatory dysfunction. Patients with restrictive ventilatory dysfunction or mixed ventilatory dysfunction were excluded from the analyses.

Ventilatory function was classified according to the 2014 Chinese national guideline for pulmonary function testing [23]. FEV_1_/VC %pred <92% was used to define abnormal ventilatory function and normal ventilation was defined as FEV_1_/VC %pred ≥ 92%. Restrictive ventilatory dysfunction was defined as FEV_1_/VC %pred ≥92% and a TLC <80%. Mixed ventilatory dysfunction was defined as FEV_1_/VC %pred <92% and TLC%pred <80%. Obstructive ventilatory dysfunction was defined as FEV_1_/VC %pred <92% and TLC %pred ≥ 80%. Small airway dysfunction (SAD) was diagnosed if at least two of the following parameters were ≤ 65% of the predicted values: FEF_50_, FEF_75,_ and FEF_25-75_ [24]. Mild airflow obstruction was defined as FEV_1_/VC %pred <92% and FEV_1_%pred ≥ 70%. Moderate to extreme airflow obstruction was defined as FEV_1_/VC %pred <92% and FEV_1_%pred <70%.

Patients who underwent BPT were included in the analysis of AHR. A Positive BPT was defined as the AHR group. Patients with negative BPT result were grouped into the normal airway responsiveness (n-AHR) group. Patients with mixed ventilatory dysfunction and restrictive ventilatory dysfunction were excluded from this analysis.

For analyses stratified by FeNO, FeNO_50_ was categorized into low (<25 ppb), middle (between 25 ppb and 50 ppb), and high (≥50 ppb) groups [17], FeNO_200_ was divided into low (<10 ppb) and high (≥10 ppb) groups and CaNO was divided into low (<5 ppb) and high (≥5 ppb) CaNO groups [25,26].

### 2.4. Statistical methods

All statistical analyses were performed using SPSS 27.0 software (SPSS Inc., IL, USA). First, frequencies, percentages, means and standard deviations were used to describe the demographic and clinical characteristics of the patients. Second, for group comparisons, Student’s *t* test or one-way analysis of variance (ANOVA) was used for normally distributed continuous variables, the Mann–Whitney *U* test or Kruskal–Wallis *H* test was used for nonnormally distributed continuous variables, and the chi-square test or Fisher’s exact test was used for dichotomous variables. Third, to test the relationships between FeNO_50_, FeNO_200,_ and CaNO and lung function parameters in patients with chronic cough, we used Pearson’s correlation for normally distributed data and Spearman’s rank correlation for nonnormally distributed data. Fourth, to determine the ability of FeNO_50_, FeNO_200_, CaNO, and lung function parameters alone to predict AHR, receiver operating characteristic (ROC) curves were used to determine biomarkers, and the best cutoff values for these parameters yielded the highest sum of diagnostic sensitivity and specificity. For joint diagnosis, we first calculated the predicted probability of the combination of exhaled nitric oxide and lung function parameters through binary logistic regression, and then constructed a ROC curve and calculated the area under the curve (AUC) based on the predicted probability.

## 3. Results

### 3.1. Subject characteristics

A total of 2205 patients with chronic cough who underwent both a spirometry test and multi-flow fractional exhaled nitric oxide measurements from January 2021 to May 2022 were included in this study. Of the included patients, 1076 (48.80%) were males. Based on the spirometry test, the population was divided into subgroups as follows: 1) normal ventilation without small airway dysfunction (*n* = 880, N + n-SAD); 2) normal ventilation with small airway dysfunction (*n* = 399, N + SAD); 3) mild airflow obstruction (*n* = 542); 4) moderate to extreme airflow obstruction (*n* = 193); 5) restrictive ventilatory dysfunction (*n* = 114); and 6) mixed ventilatory dysfunction (*n* = 77). For the stratified fractional exhaled nitric oxide groups, low FeNO_50_ (<25 ppb) accounted for 61.22%, low FeNO_200_ (<10 ppb) accounted for 37.78%, and low CaNO (<5 ppb) accounted for 40.00% of the study population (Table 1; Figure S1 in the supporting information). Patients who had normal ventilation or airflow obstruction were included in the following analyses of this study (*n* = 2014).

**Table 1.** Demographic and clinical characteristics of the study population

Characteristic	Total	Female	Male
*N* (%)	2205 (100.00)	1129 (51.20)	1076 (48.80)
Age, years	49.82 ± 16.08	47.33 ± 15.50	52.44 ± 16.27
BMI, kg/m^2^	23.86 ± 3.58	23.46 ± 3.51	24.28 ± 3.60
Lung function status			
Normal ventilation without SAD	880 (39.91)	488 (43.23)	392 (36.43)
Normal ventilation with SAD	399 (18.10)	243 (21.51)	156 (14.50)
Mild airflow obstruction	542 (24.58)	295 (26.1)	247 (22.96)
Moderate to extreme airflow obstruction	193 (8.75)	60 (5.28)	133 (12.36)
Restrictive ventilatory dysfunction	114 (5.17)	27 (2.39)	87 (8.09)
Mixed ventilatory dysfunction	77 (3.49)	16 (1.42)	61 (5.67)
FeNO_50_ (*n,* %)			
<25 ppb	1350 (61.22)	736 (65.19)	614 (57.06)
25–50 ppb	570 (25.85)	260 (23.03)	310 (28.81)
≥50 ppb	285 (12.93)	133 (11.78)	152 (14.12)
FeNO_200_ (*n,* %)			
<10 ppb	833 (37.78)	499 (44.20)	334 (31.04)
≥10 ppb	1372 (62.22)	630 (55.80)	742 (68.96)
CaNO, (*n,* %)			
<5 ppb	882 (40.00)	524 (46.41)	358 (33.27)
≥5 ppb	1323(60.00)	605 (53.58)	718 (66.73)

*Note*. Values are presented as the mean ± standard deviation for continuous variables and number (percentage) for categorical variables.

BMI, body mass index; SAD, small airway dysfunction; n-SAD, normal small airway function; ppb, parts per billion.

### 3.2. Fractional exhaled nitric oxides in patients with different airway function types

**Figure 1.**
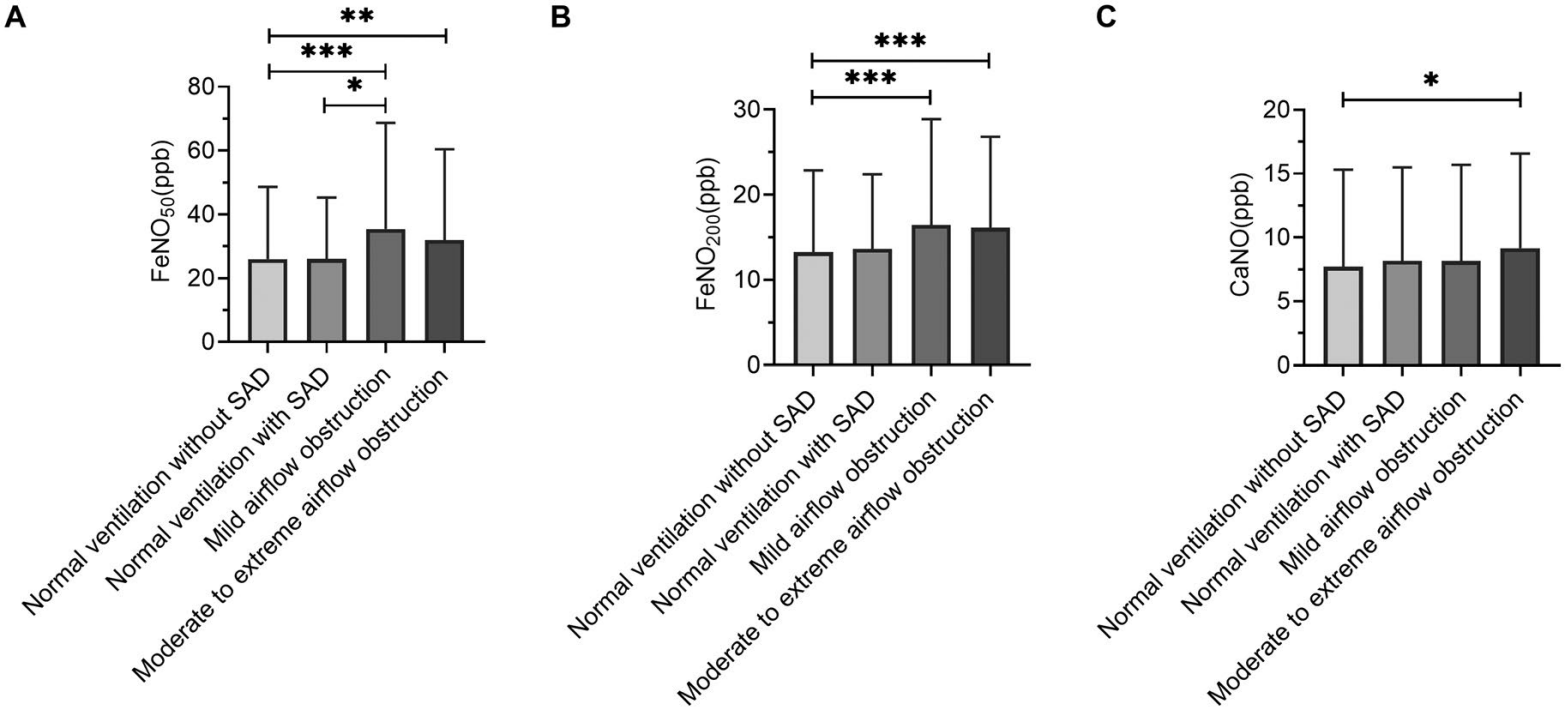
Levels of FeNO_50_, FeNO_200_ and CaNO among different airway function types. (A) Comparison of FeNO_50_ levels among patients with different airway function. The mean levels of FeNO_50_ in four groups (from left to right) were 25.93, 26.03, 35.43 and 31.95 ppb, respectively. (B) Comparison of FeNO_200_ levels among patients with different airway function. The mean levels of FeNO_200_ in four groups (from left to right) were 13.24, 13.63, 16.43 and 16.11 ppb, respectively. (C) Comparison of CaNO levels among patients with different airway function. The mean levels of CaNO in four groups (from left to right) were 7.70, 8.17, 8.16 and 9.17 ppb, respectively. *Note*: *P < 0.05; **P < 0.01; ***P < 0.001.

We first compared the levels of FeNO_50_, FeNO_200,_ and CaNO in patients with different airway function types. Compared with those in the N + n-SAD group, FeNO_50_, FeNO_200,_ and CaNO levels were all elevated in the moderate to extreme airflow obstruction group. Significant differences in FeNO_50_ and FeNO_200_ levels were also observed between the N + n-SAD group and the mild airflow obstruction group (*p* < 0.05). However, no significant differences in FeNO_50_, FeNO_200,_ or CaNO were found between the mild airflow obstruction group and the moderate to extreme airflow obstruction group (Figure 1).

Considering the impact of demographic factors on FeNO, we compared FeNO_50_, FeNO_200_, and CaNO levels among airway function types, with patients stratified by sex and by height, weight, and BMI quartiles. Within the N + n-SAD and N + SAD groups, FeNO_50_, FeNO_200_, and CaNO levels were significantly lower in females than in males. In contrast, no significant sex-based differences were observed in the moderate to severe airflow obstruction group (Figure S2 in the supporting information). In the N + n-SAD group, FeNO_50_, FeNO_200,_ and CaNO increased sequentially from the first to the fourth quartiles of both height and weight. This trend was not evident in the other airway function groups, where levels showed minimal variation across height and weight subgroups (Figures S3 and S^4^ in the supporting information). No significant differences in FeNO_50_, FeNO_200_, or CaNO levels were found across BMI quartiles in any of the airway function groups.

### 3.3. Lung function parameters in patients with varying degrees of fractional nitric oxide subgroups

**Figure 2.**
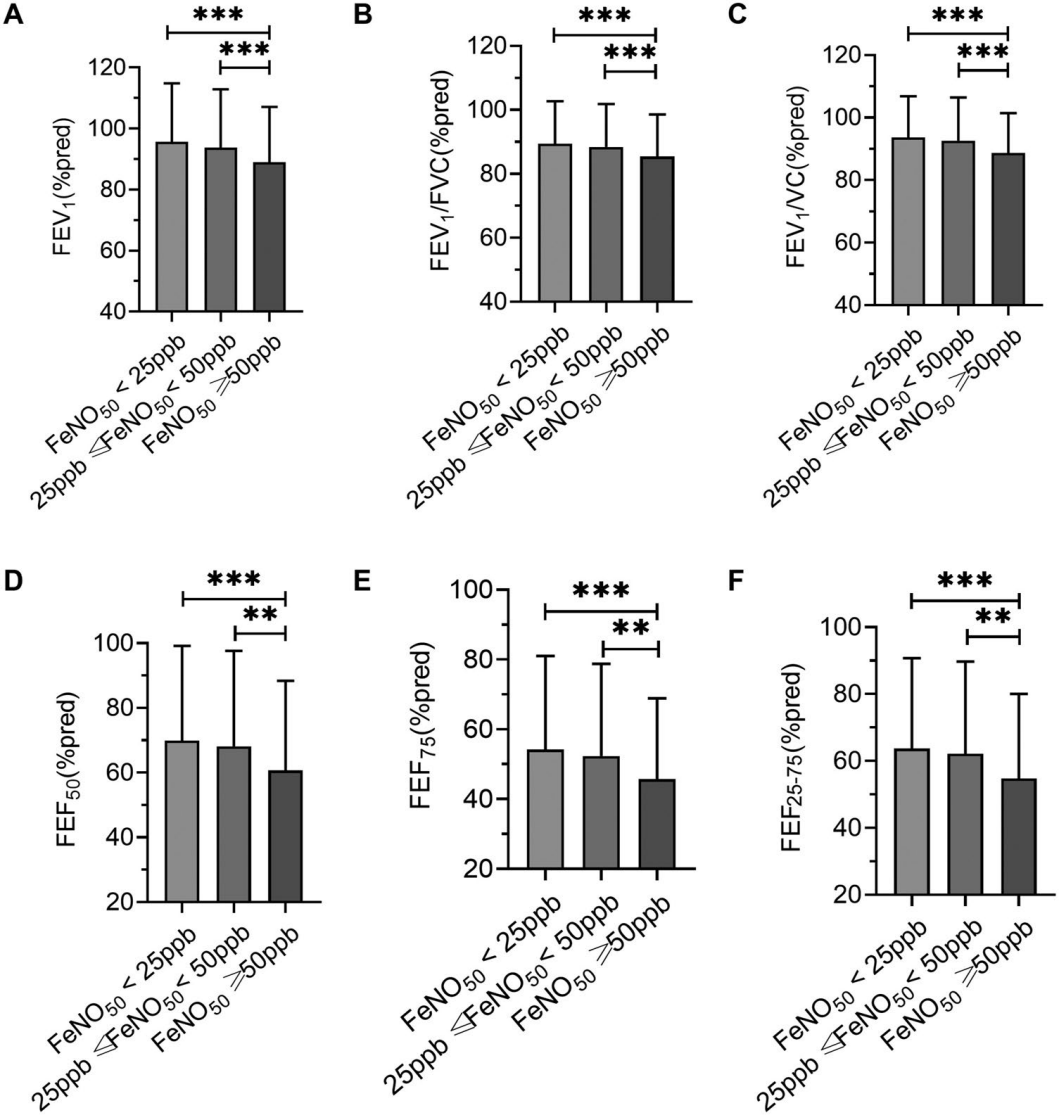
Levels of lung function parameters in stratified FeNO_50_ groups. (A–C) Levels of parameters reflecting large airway function (the predicted value of FEV_1_, FEV_1_/FVC, FEV_1_/VC) in the low FeNO_50_ (<25 ppb), middle FeNO_50_ (25–50 ppb) and high FeNO_50_ (≥50 ppb) groups. (D–F) Levels of parameters reflecting small airway function (the predicted value of FEF_50_, FEF_75_, FEF_25-75_) in the low FeNO_50_ (<25 ppb), middle FeNO_50_ (25–50 ppb) and high FeNO_50_ (≥50 ppb) groups. *Note*: *P < 0.05; **P < 0.01; ***P < 0.001.

To assess airway function across varying degrees of airway T2 inflammation, we compared lung function parameters among stratified FeNO_50_, FeNO_200,_ and CaNO subgroups (*n* = 2014). Compared to the low FeNO_50_ group (<25 ppb, *n* = 1226), both the large airway (FEV_1_%pred, FEV_1_/FVC %pred, FEV_1_/VC %pred) and the small airway (FEF_50_%pred, FEF_75_%pred_,_ FEF_25–75_%pred) parameters were significantly lower in the middle FeNO_50_ (25–50 ppb, *n* = 518) and the high FeNO_50_ (≥50 ppb, *n* = 270) groups. Notably, significant differences in these parameters were also observed between the middle and high FeNO_50_ groups (Figure 2; Table S1 in the supporting information).

**Figure 3.**
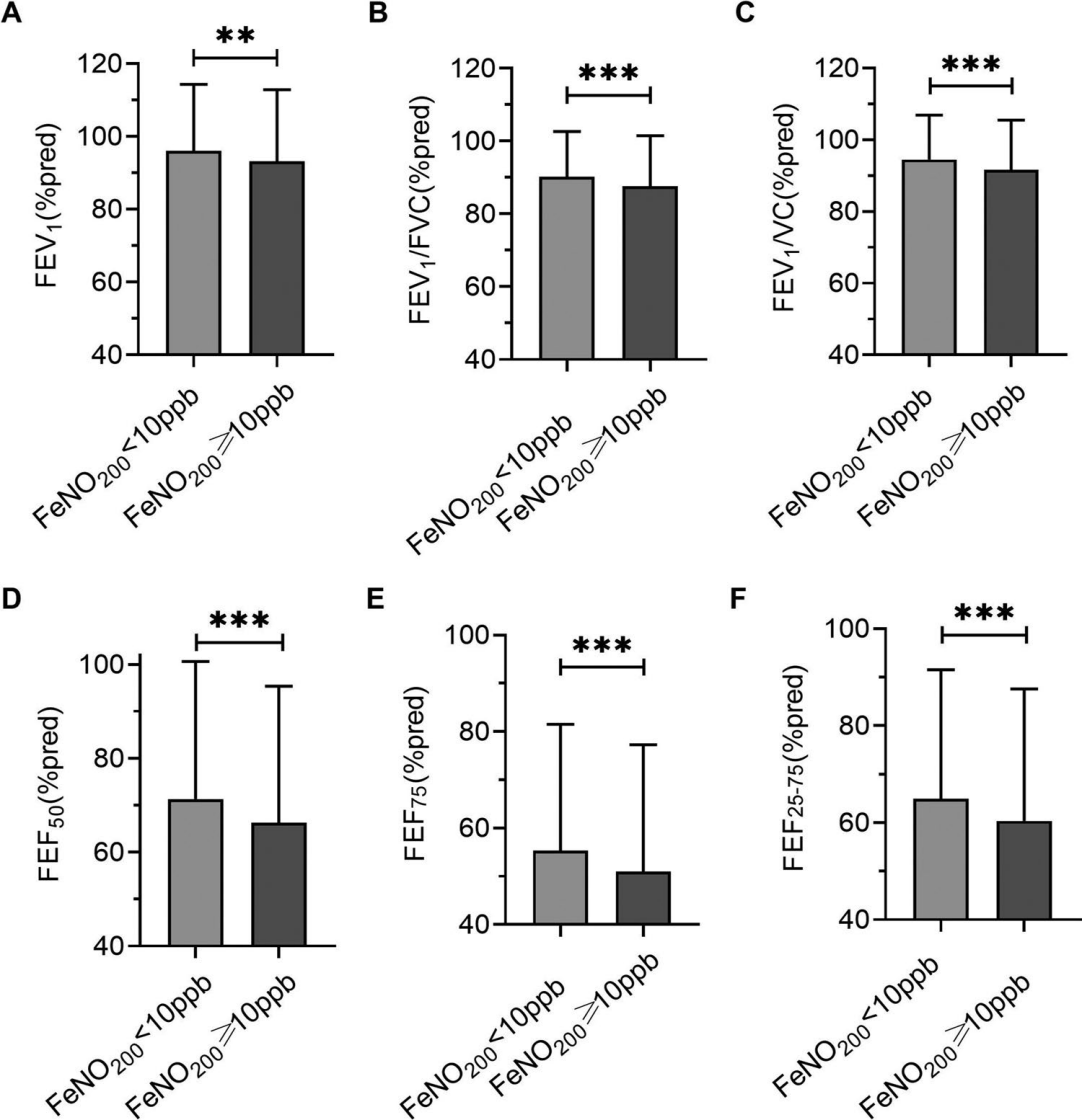
Levels of lung function parameters in stratified FeNO_200_ groups. (A–C) Levels of parameters reflecting large airway function (the predicted values of FEV_1_, FEV_1_/FVC, FEV_1_/VC) in the low FeNO_200_ (<10 ppb) and high FeNO_200_ (≥10 ppb) groups. (D–F) Levels of parameters reflecting small airway function (the predicted values of FEF_50_, FEF_75_, FEF_25-75_) in the low FeNO_200_ (<10 ppb) and high FeNO_200_ (≥10 ppb) groups. *Note*. *P < 0.05; **P < 0.01; ***P < 0.001.

**Figure 4.**
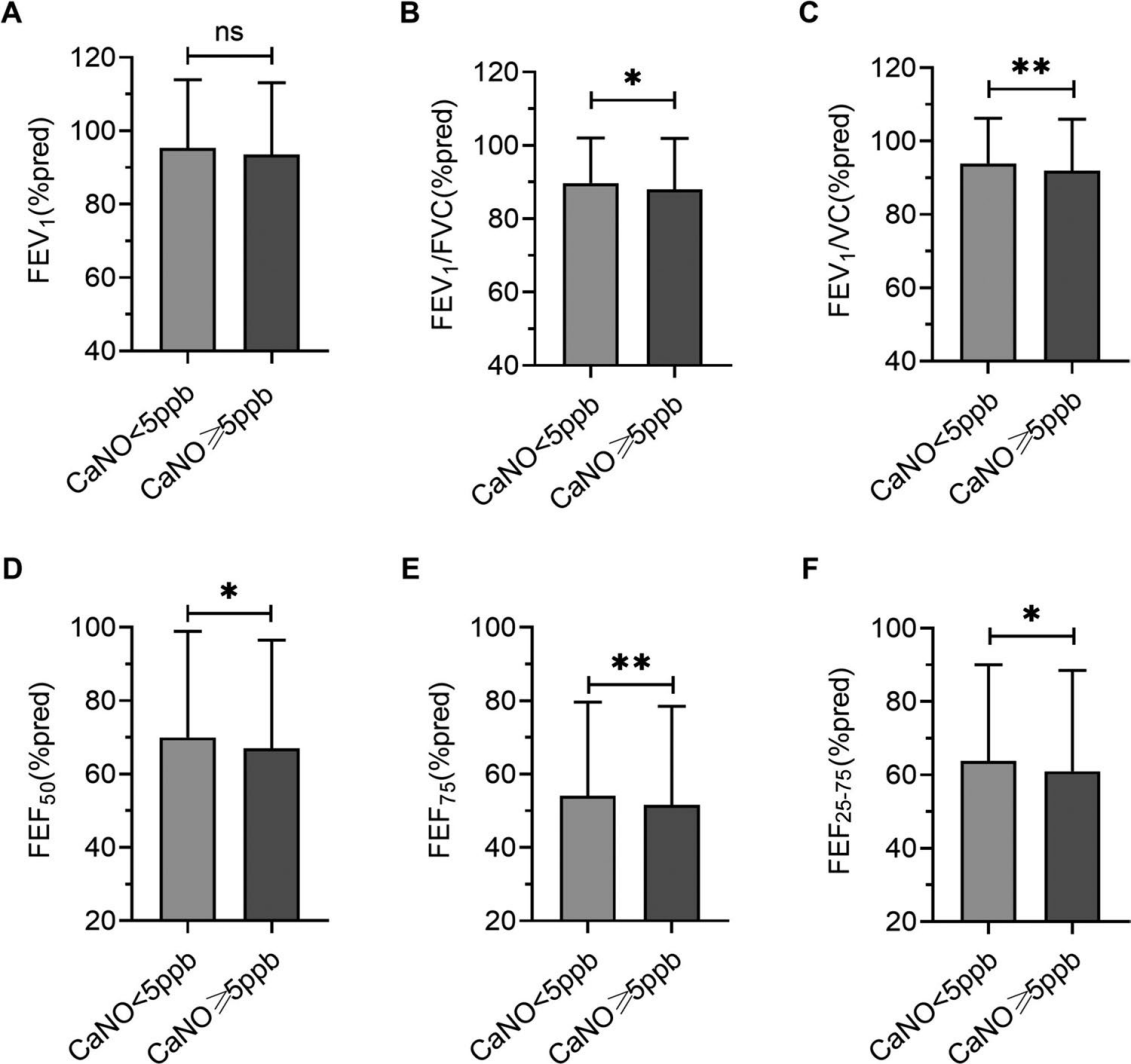
Levels of lung function parameters in stratified CaNO groups. (A–C) Levels of parameters reflecting large airway function (the predicted values of FEV_1_, FEV_1_/FVC, FEV_1_/VC) in the low CaNO (<5 ppb) and high CaNO (≥5 ppb) groups. (D–F) Levels of parameters reflecting small airway function (the predicted values of FEF_50_, FEF_75_, FEF_25-75_) in the low CaNO (<5 ppb) and high FeNO_200_ (≥5 ppb) groups. *Note*. *P < 0.05; **P < 0.01; ***P < 0.001.

Similarly, both the large airway and the small airway parameters were lower in the high FeNO_200_ group (≥10 ppb, *n* = 1249) compared with the low FeNO_200_ group (<10 ppb, *n* = 765) (Figure 3; Table S2 in the supporting information). For CaNO, the high group (≥5 ppb, *n* = 1210) had significantly lower predicted values for FEV_1_/FVC, FEV_1_/VC, FEF_50_, FEF_75_, and FEF_25–75_ compared to the low group (<5 ppb, *n* = 804). However, no significant difference in FEV_1_ (%pred) was found between CaNO subgroups (Figure 4; Table S3 in the supporting information).

To evaluate sex-based differences in airway function across type 2 inflammation subgroups, we compared lung function parameters between females and males within each FeNO_50_, FeNO_200_, and CaNO subgroup. In the N + n-SAD population, females in the low and middle FeNO_50_ subgroups exhibited significantly higher predicted values for FEV_1_, FEV_1_/VC, FEF_75_, and FEF_25–75_ than their male counterparts (Table S4 in the supporting information). In contrast, no significant sex differences in these parameters were observed within the N + SAD or mild airflow obstruction groups. Interestingly, the same trend was noted in the moderate to severe airflow obstruction group, where females showed higher predicted values for FEV_1_/FVC, FEV_1_/VC, FEF_50_, and FEF_25–75_ than males (Tables S5–S7 in the supporting information). However, the absolute magnitude of these sex-specific differences across all subgroups was small. Given the minimal clinical magnitude of these differences, no consistent or conclusive pattern regarding the impact of sex on lung function within airway T2 inflammation subgroups can be established.

### 3.4. Relationships between exhaled nitric oxide levels and lung function parameters

Furthermore, Spearman correlation analyses across the entire cohort (*n* = 2014) showed that FeNO_50_ and FeNO_200_ levels were significantly negatively correlated with both large and small airway function parameters. Although CaNO also showed some significant correlations, its coefficients were consistently weaker (all <0.1) than those for FeNO_50_ and FeNO_200_ (Figure S5; Table S8 in the supporting information).

Correlation analyses stratified by sex revealed that these inverse correlations with FeNO_50_ and FeNO_200_ were generally stronger in females (*n* = 1089) than in males (*n* = 925) (Tables S9 and S^10^ in the supporting information). When further stratified by both airway function type and sex, significant associations were rarely observed across the subgroups (Tables S11–S18 in the supporting information). Despite achieving statistical significance in some analyses, all correlation coefficients were weak (approximately 0.1), suggesting minimal clinical relevance. These findings require cautious interpretation and warrant further investigation.

### 3.5. Diagnostic accuracy of FeNO_50_, FeNO_200_, CaNO, and lung function parameters for AHR prediction

To assess the ability of FeNO_50_, FeNO_200_, CaNO, and lung function parameters to diagnose AHR among outpatients with chronic cough, we included 582 chronic cough outpatients who underwent methacholine BPT. Of these, 156 were AHR-positive and 426 were AHR-negative (n-AHR). Compared with the non-AHR group, AHR patients had significantly higher FeNO_50_ and FeNO_200_ levels and poorer lung function, whereas CaNO levels did not differ (Table S19 in the supporting information). In this AHR-prediction subgroup, FeNO_50_ and FeNO_200_ remained negatively correlated with lung function parameters, while CaNO showed no significant correlations (Table S20; Figure S6 in the supporting information).

**Figure 5.**
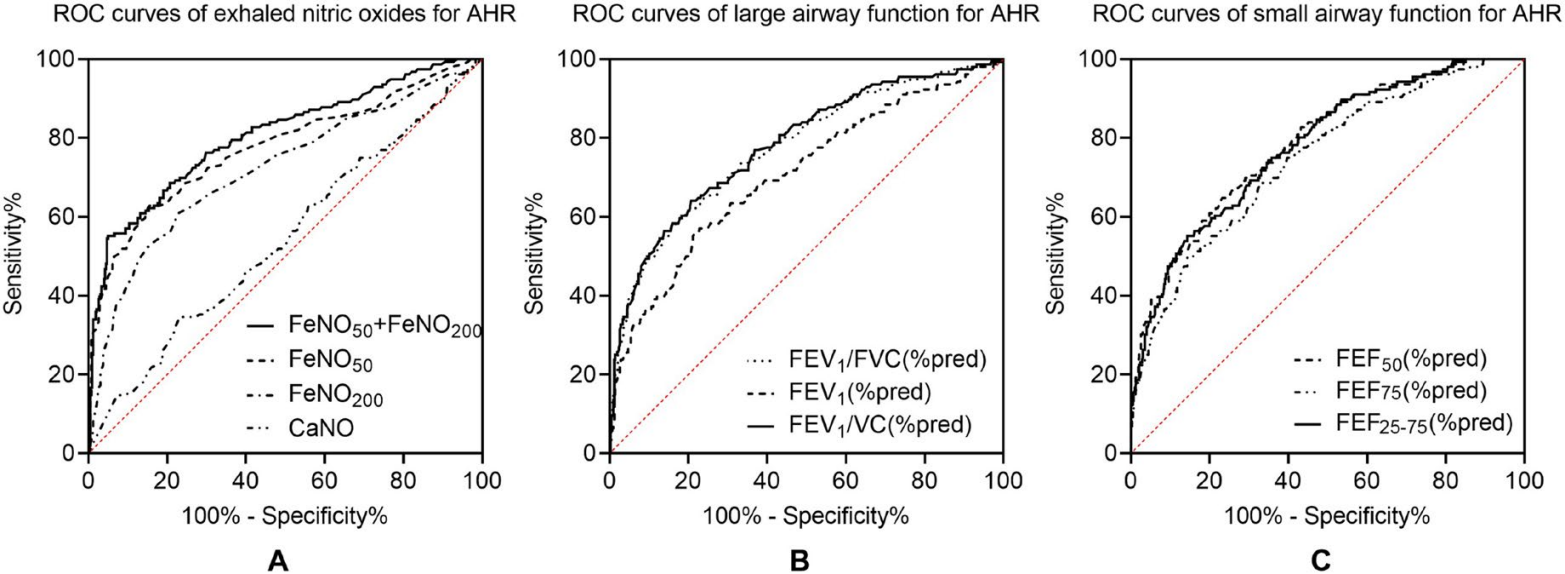
ROC curves of exhaled nitric oxide and lung parameters of large airway and small airway for predicting AHR. (A) ROC curves of FeNO_50_, FeNO_200_, CaNO and the joint model of FeNO_50_ and FeNO_200_ for predicting AHR. AUC (FeNO_50_) = 0.775 (95% CI: 0.727–0.823); AUC (FeNO_200_) = 0.721 (95% CI: 0.670–0.771); AUC (FeNO_50_ + FeNO_200_) = 0.805 (95% CI: 0.762–0.849); Cutoff (FeNO_50_) = 31.5ppb; Cutoff (FeNO_200_) = 14.5ppb. (B) ROC curves of lung parameters reflecting large airway function (the predicted value of FEV_1_, FEV_1_/FVC, FEV_1_/VC) for predicting AHR. AUC [FEV_1_(%pred)] = 0.708 (95% CI: 0.658–0.785); AUC [(FEV_1_/FVC(%pred)] = 0.775 (95% CI: 0.730–0.819); AUC [FEV_1_/VC(%pred)] = 0.782 (95% CI: 0.738–0.826); cutoff [FEV_1_(%pred)] = 91.55%; Cutoff [(FEV_1_/FVC(%pred)] = 88.05%; Cutoff [FEV_1_/VC(%pred)] = 93.15%. (C) ROC curves of lung parameters reflecting small airway function (the predicted value of FEF_50_, FEF_75_, FEF_25-75_) for predicting AHR. AUC [FEF_50_(%pred)] = 0.785 (95%CI: 0.744–0.827); AUC [FEF_75_(%pred)] = 0.745 (95%CI: 0.700,0.790); AUC [FEF_25-75_(%pred)] = 0.780 (95%CI: 0.738–0.822); Cutoff [FEF_50_(%pred)] = 64.05%; Cutoff [(FEF_75_(%pred)] = 40.95%; Cutoff [FEF_25-75_(%pred)] = 53.25%. Abbreviation: ROC, receiver operator characteristic; AUC, area under the curve; CI, confidence interval.

FeNO_50_ and FeNO_200_ showed moderate predictive ability for AHR in patients with chronic cough. The AUCs of FeNO_50_ and FeNO_200_ for predicting AHR were 0.775 (95% CI: 0.727–0.823) and 0.721 (95% CI: 0.670–0.771), respectively. The optimal cutoff value of FeNO_50_ was 31.5 ppb, with a sensitivity of 62.8% and a specificity of 84.5%. The optimal cutoff point of FeNO_200_ was 14.5 ppb, with a sensitivity of 60.9% and a specificity of 77.2%. However, CaNO failed to distinguish between outpatients in the AHR group and the n-AHR group (Figure 5; Table S21 in the supporting Information).

The AUCs of the predicted FEV_1_, FEV_1_/FVC, FEV_1_/VC, FEF_50_, FEF_75,_ and FEF_25-75_ for predicting AHR all reached the threshold for statistical significance. The largest AUC for a positive AHR diagnosis was for FEF_50_(%pred) [0.785 (95% CI: 0.744–0.827)], with a sensitivity of 64.7% and a specificity of 76.8% (Figure 5; Table S21 in the supporting information).

To determine whether combined measurements improve AHR prediction, we repeated the ROC analysis using paired combinations of exhaled nitric oxide and lung function parameters. All AUCs in paired combinations were greater than those in single measurements (Table S22 in the supporting information). The AUC of FeNO_50_ combined with FeNO_200_ was 0.805 (95% CI: 0.762–0.849), with a low sensitivity of 55.1% and a high specificity of 85.1%. Binary logistic regression of the predicted values of FeNO_50_ and FEF_50_ (%pred) produced the highest AUC of 0.895 (95% CI: 0.865–0.925), with a sensitivity of 85.9% and specificity of 80.8%.

## 4. Discussion

This large-scale retrospective study provides real-world evidence on the relationships between multi-flow FeNO parameters and lung function in patients with chronic cough. We found that FeNO_50_ and FeNO_200_ levels are significantly elevated in patients with airflow obstruction and showed a negative association with both large and small airway function parameters.. Furthermore, FeNO_50_ or FeNO_200_ alone demonstrated moderate predictive value for AHR, which is significantly enhanced when combined with spirometric indices—most effectively with FeNO_50_ and FEF_50_ (%pred) (AUC: 0.895). In contrast, CaNO shows minimal association with lung function and no diagnostic utility for AHR in this study.

FeNO_50_ is widely used in asthma management [12,27]. Its concentration is determined by both inflammatory activity and airway caliber, studies with airway challenges demonstrated that a reduction in airway caliber decreased FeNO_50_ levels [28–30]. This study demonstrated that FeNO_50_ and FeNO_200_ are higher in patients with worse airflow obstruction regardless of the causes of chronic cough.

Factors such as sex, height, and environmental factors were associated with the reference values of FeNO_50_ [31–35]. In accordance with published studies, we concluded that the FeNO_50_, FeNO_200,_ and CaNO concentrations of females were significantly lower than those of males. Height and weight, but not BMI, were influencing factors of FeNO_50_, FeNO_200,_ and CaNO in chronic cough patients with both normal ventilation and small airway function. However, the impacts of demographic factors on exhaled nitric oxide levels disappeared in patients with worsening airflow obstruction, indicating that the dominant role of airway inflammation and demographic factors influencing FeNO values was limited to individuals with normal airway function.

Most studies have reported negative correlations between FeNO_50_ and airway function in patients with asthma or other obstructive diseases. CaNO was significantly correlated with FEF_25/75_ (%pred), FEF_50_ (%pred), and FEV_1_/FVC (%pred) in patients with asthma [36,37]. In this study, the negative relationships between the concentrations of FeNO_50_, FeNO_200_, and CaNO and lung function parameters of both large and small airways were statistically significant in outpatients with chronic cough. While statistically significant, all observed correlations between FeNO and lung function parameters were weak (coefficients ∼0.1). Therefore, these correlations of ∼0.1 indicate FeNO explains only a small fraction of the variance in lung function. Thus, this precludes a conclusion of strong predictive power for airflow obstruction. In the heterogeneous population of chronic cough—where etiology spans from upper airway cough syndrome to gastroesophageal reflux—FeNO cannot and should not be used as a standalone, quantitative predictor of the degree of airflow obstruction in an individual patient. They help identify a subgroup of patients in whom T2 inflammation is a more likely contributor to symptoms, thereby prioritizing them for further investigations like bronchoprovocation testing or a therapeutic trial with anti-inflammatory medications.

AHR is a characteristic feature of asthma [38], and various studies have suggested that FeNO_50_ and small airway parameters alone or in combination might be surrogate markers for predicting AHR [13,39–42]. Yi et al. concluded that a FeNO_50_ of 31.5 ppb had a low sensitivity (54.0%) and high specificity (91.4%) in predicting corticosteroid-responsive cough from chronic cough [42]. Similarly, the cutoff value of FeNO_50_ for AHR in our cohort was 31.5 ppb, with a low sensitivity of 62.8% and a high specificity of 84.5%. Bai et al. reported that both FeNO_200_ (>11 ppb) and CaNO (>3.6 ppb) were useful markers for differentiating cough variant asthma from chronic cough, and the AUCs of FeNO_200_ combined with three small airway parameters were greater than those of FeNO_50_ for the diagnosis of CVA [43]. In this study, the FeNO_200_ concentration (>14.5 ppb) could also predict AHR with a high specificity of 77.2% and a relatively low sensitivity of 60.9% in patients with chronic cough. Additionally, we demonstrated that worse large airway function parameters (the predicted values of FEV_1_, FEV_1_/FVC, and FEV_1_/VC) or small airway function parameters (the predicted values of FEF_50_, FEF_75,_ and FEF_25–75_) were also associated with a greater likelihood of AHR, with lower sensitivity and higher specificity. Various studies have reported that the joint models of FeNO_50_ and small airway function parameters increase the AUC for AHR [13,41,44,45]. In this study, we further investigated the ability of large airway function parameters to predict AHR. FeNO_50_ or FeNO_200_, combined with lung function parameters of either large or small airways produced higher AUCs than that of the joint model of FeNO_50_ and FeNO_200_ (AUC, 0.805). Combining FeNO_50_ (>31.5 ppb) and FEF_50_ (%pred, <64.5%) provided a better prediction of AHR than did the other paired combinations, allowing clinicians to differentiate patients with AHR from outpatients reporting chronic cough.

Inconsistently, CaNO was dissociated from lung function and failed to predict AHR in this study. Methodologically, the calculation of CaNO using flow rates of 50 and 200 mL/s is based on established models recommended by Chinese guideline (No. PREPARE-2024CN685) but differs from the 2017 ERS technical standard which recommends using flows ≥100 mL/s [46]. This difference may affect the accuracy and absolute values of CaNO, potentially limiting direct comparability with studies adhering strictly to the ERS standard. Pathophysiologically, the poor performance of CaNO may indicate that in a broad respiratory clinic cohort of chronic cough, the dominant inflammatory signal originates from the proximal (conducting) airways, robustly captured by FeNO_50_ and FeNO_200_, rather than from the alveolar compartment. Furthermore, as AHR is primarily a functional phenomenon of the conducting airways, its weak link with CaNO is perhaps not surprising. This suggests CaNO may have limited additive value in the initial assessment of unselected chronic cough. This result may be because the standards of positive BPT were based on FEV1 decline and airway constrictions were limited to conducting airways, while the CaNO concentration may not be affected by bronchoconstriction [47].

Several limitations inherent in our study design should be considered when interpreting the findings. First, the retrospective, single-center study design may introduce selection bias and limit the generalizability of our results. Second, the FeNO measurement methods followed the specifications of the device used, with exhalation times of ≥4 s (50 mL/s) and ≥2 s (200 mL/s). While this is the validated protocol for the SUNVOU-CA2122 analyzer, these durations are shorter than the 10-s exhalation recommended in some guidelines to minimize upper airway NO contamination. This represents a potential technical limitation, especially for the interpretation of alveolar NO (CaNO) values. Third, the lack of systematically collected data on key potential confounders—including detailed smoking history, atopic status, recent use of corticosteroids or montelukast, and history of upper respiratory infections—prevents adjustment for these factors, which are known to influence FeNO levels. Although patients with recent infections or medication use were typically not referred for FeNO testing, the absence of these data remains a constraint on causal inference. Fourth, the studied cohort comprised a heterogeneous population of chronic cough patients without sub-phenotyping by definitive etiology (e.g. cough-variant asthma, UACS). This heterogeneity likely dilutes associations that might be more pronounced in specific clinical subgroups. In addition, normal ventilatory function was classified using FEV_1_/VC %pred ≥92%, according to the 2014 Chinese national pulmonary function testing guideline and the routine clinical reporting standard used in our clinical setting. Therefore, our findings should be interpreted in this context, and future studies using the Global Lung Function Initiative (GLI)-based classification are warranted to confirm our results. Finally, the cross-sectional analysis establishes association but precludes any determination of causality between FeNO levels and longitudinal lung function changes. Prospective, longitudinal studies in well-characterized cohorts are needed to elucidate these causal pathways.

## 5. Conclusion

This study demonstrates that in patients with chronic cough, elevated FeNO_50_ and FeNO_200_ levels are associated with worse airflow limitation and lung function, and offer moderate predictive value for airway hyperresponsiveness (AHR), with optimal cut-off values of 31.5 ppb and 14.5 ppb, respectively. Combining FeNO with lung function parameters, particularly FEF_50_ (%pred), significantly improves predictive performance. However, the observed correlations were overall weak, indicating that FeNO may be interpreted as a risk-stratification biomarker rather than a standalone diagnostic tool for quantifying airflow obstruction. In contrast, CaNO did not provide meaningful diagnostic utility in this cohort. While the limitations inherent in this retrospective analysis warrant cautious interpretation, our findings underscore the clinical utility of these specific exhaled nitric oxide parameters. Further prospective studies are warranted to validate their role in guiding personalized management.

## Supplementary Material

Supplemental Material

## Data Availability

The data used and analyzed in the current study are available from the corresponding author on reasonable request.

## Abbreviations

FeNO_50_: Fractional exhaled nitric oxide at a flow rate of 50mL/s
FeNO_200_: Fractional exhaled nitric oxide at a flow rate of 200mL/s
CaNO: The alveolar concentration of fractional exhaled nitric oxide
AHR: Airway hyperresponsiveness
AUC: Area under the curves
ROC: Receiver operating characteristic
ATS: American Thoracic Society
ERS: European Respiratory Society
FEV_1_ (%pred): Forced expiratory volume in 1 second of predicted value
FVC (%pred): Forced vital capacity of predicted value
VC (%pred): Vital capacity of predicted value
FEV_1_/FVC(%pred): Forced expiratory volume in 1 second to forced vital capacity ratio of predicted value
FEV_1_/VC (%pred): Forced expiratory volume in 1 second to vital capacity ratio of predicted value
FEF_50_ (%pred): Forced expiratory flow at 50% of forced vital capacity of predicted value
FEF_75_ (%pred): Forced expiratory flow at 75% of forced vital capacity of predicted value
FEF_25–75_ (%pred): Forced expiratory flow between 25% and 75% of predicted value
TLC (%pred): Total lung capacity of predicted value
BPT: Bronchoprovocation test
BDT: Bronchodilation test
SAD: Small airway dysfunction

## References

[1] Yang X, Hu M, Huang K. Demographics, risk factors and prevalence of chronic cough in Asian general adult population: a narrative review. J Thorac Dis. 2025;17(5):3419–3432. doi: 10.21037/jtd-24-248. 40529737 PMC12170037

[2] Rouadi PW, Idriss SA, Bousquet J, et al. WAO–ARIA consensus on chronic cough: executive summary. World Allergy Organ J. 2025;18(3):101034. doi: 10.1016/j.waojou.2025.101034. 40093560 PMC11903822

[3] Chinese Thoracic Society. Chinese Medical Association. [Guidelines for the prevention and management of bronchial asthma. Zhonghua Jie He He Hu Xi Za Zhi. 2025;48(3):208–248. 40050074 10.3760/cma.j.cn112147-20241013-00601

[4] Michils A, Haccuria A, Virreira M, et al. How airway caliber affects FeNO thresholds used to identify type 2 inflammation in asthma. J Allergy Clin Immunol Pract. 2025;13(9):2397–2404. doi: 10.1016/j.jaip.2025.05.049. 40480547

[5] Çolak Y, Afzal S, Marott JL, et al. Type-2 inflammation and lung function decline in chronic airway disease in the general population. Thorax. 2024;79(4):349–358. doi: 10.1136/thorax-2023-220972. 38195642 PMC10958305

[6] Petousi N, Pavord ID, Kent BD. Type-2 inflammation: a key treatable trait associated with lung function decline in chronic airways disease. Thorax. 2024;thorax:2023–221329. doi: 10.1136/thorax-2023-221329. 38373823

[7] Matsunaga K, Hirano T, Oka A, et al. Persistently high exhaled nitric oxide and loss of lung function in controlled asthma. Allergol Int. 2016;65(3):266–271. doi: 10.1016/j.alit.2015.12.006. 26822895

[8] Coumou H, Westerhof GA, de Nijs SB, et al. Predictors of accelerated decline in lung function in adult-onset asthma. Eur Respir J. 2018;51(2):1701785. doi: 10.1183/13993003.01785-2017. 29444915

[9] Högman M, Janson C, Palm A, et al. Exhaled nitric oxide stability over two years in relation to COPD outcomes. J Breath Res. 2025;19(4):046005. doi: 10.1088/1752-7163/adfd04. 40829619

[10] Hirano T, Matsunaga K, Sugiura H, et al. Relationship between alveolar nitric oxide concentration in exhaled air and small airway function in COPD. J Breath Res. 2013;7(4):046002. doi: 10.1088/1752-7155/7/4/046002. 24091810

[11] Högman M, Palm A, Sulku J, et al. Alveolar nitric oxide in chronic obstructive pulmonary disease – a two-year follow-up. Biomedicines. 2022;10(9):2212. doi: 10.3390/biomedicines10092212. 36140313 PMC9496546

[12] Song W-J, Kim HJ, Shim J-S, et al. Diagnostic accuracy of fractional exhaled nitric oxide measurement in predicting cough-variant asthma and eosinophilic bronchitis in adults with chronic cough: a systematic review and meta-analysis. J Allergy Clin Immunol. 2017;140(3):701–709. doi: 10.1016/j.jaci.2016.11.037. 28088474

[13] Bao W, Zhang X, Lv C, et al. The value of fractional exhaled nitric oxide and forced mid-expiratory flow as predictive markers of bronchial hyperresponsiveness in adults with chronic cough. J Allergy Clin Immunol Pract. 2018;6(4):1313–1320. doi: 10.1016/j.jaip.2017.09.026. 29128336

[14] Gong Z, Huang J, Xu G, et al. The value of bronchodilator response in FEV1 and FeNO for differentiating between chronic respiratory diseases: an observational study. Eur J Med Res. 2024;29(1):97. doi: 10.1186/s40001-024-01679-w. 38311782 PMC10840153

[15] Hao H, Bao W, Xue Y, et al. Spirometric changes in bronchodilation tests as predictors of asthma diagnosis and treatment response in patients with FEV1 ≥ 80% predicted. J Allergy Clin Immunol Pract. 2021;9(8):3098–3108.e4. doi: 10.1016/j.jaip.2021.03.015. 33766580

[16] Hao H, Pan Y, Xu Z, et al. Prediction of bronchodilation test in adults with chronic cough suspected of cough variant asthma. Front Med (Lausanne). 2022;9:987887. doi: 10.3389/fmed.2022.987887. 36569143 PMC9780531

[17] Dweik RA, Boggs PB, Erzurum SC, et al. An official ATS clinical practice guideline: interpretation of exhaled nitric oxide levels (FENO) for clinical applications. Am J Respir Crit Care Med. 2011;184(5):602–615. doi: 10.1164/rccm.9120-11ST. 21885636 PMC4408724

[18] Paredi P, Kharitonov SA, Meah S, et al. A novel approach to partition central and peripheral airway nitric oxide. Chest. 2014;145(1):113–119. doi: 10.1378/chest.13-0843. 23989961

[19] Zheng S, Chen S, Hu Y, et al. Alveolar nitric oxide concentration plays an important role in identifying cough variant asthma and assessing asthma control in children. J Asthma. 2024;61(4):328–337. doi: 10.1080/02770903.2023.2272806. 37855443

[20] Wang J, Wu K, Cheng X, et al. The value of concentration of alveolar nitric oxide in diagnosing small airway dysfunction in patients with stable asthma. Clin Respir J. 2023;17(5):357–363. doi: 10.1111/crj.13565. 36508744 PMC10214569

[21] Liu J, Li Z, Liu Z, et al. Exhaled nitric oxide from the central airway and alveoli in OSAHS patients: the potential correlations and clinical implications. Sleep Breath. 2016;20(1):145–154. doi: 10.1007/s11325-015-1198-7. 26084410

[22] Graham BL, Steenbruggen I, Miller MR, et al. Standardization of spirometry 2019 update: an Official American Thoracic Society and European Respiratory Society Technical Statement. Am J Respir Crit Care Med. 2019;200(8):e70–88–e88. doi: 10.1164/rccm.201908-1590ST. 31613151 PMC6794117

[23] Pulmonary Function Professional Group. Chinese Thoracic Society, Chinese Medical Association. [Guidelines for pulmonary function tests (Part II): spirometry]. Zhonghua Jie He He Hu Xi Za Zhi. 2014;37(7):481–486 (in Chinese). doi: 10.3760/cma.j.issn.1001-0939.2014.07.001.

[24] Xiao D, Chen Z, Wu S, et al. Prevalence and risk factors of small airway dysfunction, and association with smoking, in China: findings from a national cross-sectional study. Lancet Respir Med. 2020;8(11):1081–1093. doi: 10.1016/S2213-2600(20)30155-7. 32598906

[25] Delclaux C, Mahut B, Zerah-Lancner F, et al. Increased nitric oxide output from alveolar origin during liver cirrhosis versus bronchial source during asthma. Am J Respir Crit Care Med. 2002;165(3):332–337. doi: 10.1164/ajrccm.165.3.2107017. 11818316

[26] Puckett JL, Taylor RWE, Leu S-Y, et al. Clinical patterns in asthma based on proximal and distal airway nitric oxide categories. Respir Res. 2010;11(1):47. doi: 10.1186/1465-9921-11-47. 20426813 PMC2876084

[27] Rupani H, Kent BD. Using fractional exhaled nitric oxide measurement in clinical asthma management. Chest. 2022;161(4):906–917. doi: 10.1016/j.chest.2021.10.015. 34673021

[28] Haccuria A, Michils A, Michiels S, et al. Exhaled nitric oxide: a biomarker integrating both lung function and airway inflammation changes. J Allergy Clin Immunol. 2014;134(3):554–559. doi: 10.1016/j.jaci.2013.12.1070. 24522091

[29] Michils A, Akset M, Haccuria A, et al. The impact of airway obstruction on feno values in asthma patients. J Allergy Clin Immunol Pract. 2024;12(1):111–117. doi: 10.1016/j.jaip.2023.08.027. 37634805

[30] Bertolini F, Sprio AE, Baroso A, et al. Predictors of low and high exhaled nitric oxide values in asthma: a real-world study. Respiration. 2022;101(8):746–756. doi: 10.1159/000524498. 35512642

[31] Dressel H, de la Motte D, Reichert J, et al. Exhaled nitric oxide: independent effects of atopy, smoking, respiratory tract infection, gender and height. Respir Med. 2008;102(7):962–969. doi: 10.1016/j.rmed.2008.02.012. 18396030

[32] Brody DJ, Zhang X, Kit BK, et al. Reference values and factors associated with exhaled nitric oxide: U.S. youth and adults. Respir Med. 2013;107(11):1682–1691. doi: 10.1016/j.rmed.2013.07.006. 24041745

[33] Blake TL, Chang AB, Chatfield MD, et al. Does ethnicity influence fractional exhaled nitric oxide in healthy individuals? A systematic review. Chest. 2017;152(1):40–50. doi: 10.1016/j.chest.2017.02.007. 28215791

[34] Zhang X, Xu Z, Lin J, et al. Sex differences of small airway function and fractional exhaled nitric oxide in patients with mild asthma. Ann Allergy Asthma Immunol. 2023;130(2):187–198.e3. doi: 10.1016/j.anai.2022.11.010. 36400352

[35] Werthmann D, van Wendel de Joode B, Cuffney MT, et al. A cross-sectional analysis of medical conditions and environmental factors associated with fractional exhaled nitric oxide (FeNO) in women and children from the ISA birth cohort, Costa Rica. Environ Res. 2023;233:116449. doi: 10.1016/j.envres.2023.116449. 37356534 PMC10529647

[36] Fujisawa T, Yasui H, Akamatsu T, et al. Alveolar nitric oxide concentration reflects peripheral airway obstruction in stable asthma. Respirology. 2013;18(3):522–527. doi: 10.1111/resp.12031. 23240824

[37] Kobayashi D, Tochino Y, Kanazawa H, et al. Comparison of alveolar nitric oxide concentrations using two different methods for assessing small airways obstruction in asthma. Respirology. 2011;16(5):862–868. doi: 10.1111/j.1440-1843.2011.01989.x. 21564400

[38] Nair P, Martin JG, Cockcroft DC, et al. Airway hyperresponsiveness in asthma: measurement and clinical relevance. J Allergy Clin Immunol Pract. 2017;5(3):649–659.e2. doi: 10.1016/j.jaip.2016.11.030. 28163029

[39] Liu J, Xu R, Zhan C, et al. Clinical utility of ultrahigh fractional exhaled nitric oxide in predicting bronchial hyperresponsiveness in patients with suspected asthma. Postgrad Med J. 2019;95(1128):541–546. doi: 10.1136/postgradmedj-2018-136333. 31296792

[40] Malerba M, Ragnoli B, Azzolina D, et al. Predictive markers of bronchial hyperreactivity in a large cohort of young adults with cough variant asthma. Front Pharmacol. 2021;12:630334. doi: 10.3389/fphar.2021.630334. 33953671 PMC8089476

[41] Zhu H, Zhang R, Hao C, et al. Fractional exhaled nitric oxide (FeNO) combined with pulmonary function parameters shows increased sensitivity and specificity for the diagnosis of cough variant asthma in children. Med Sci Monit. 2019;25:3832–3838. doi: 10.12659/MSM.913761. 31120043 PMC6543875

[42] Yi F, Chen R, Luo W, et al. Validity of fractional exhaled nitric oxide in diagnosis of corticosteroid-responsive cough. Chest. 2016;149(4):1042–1051. doi: 10.1016/j.chest.2016.01.006. 26836931

[43] Bai H, Shi C, Yu S, et al. A comparative study on the value of lower airway exhaled nitric oxide combined with small airway parameters for diagnosing cough-variant asthma. Ther Adv Respir Dis. 2023;17:17534666231181259. doi: 10.1177/17534666231181259. 37326344 PMC10278400

[44] Bao W, Zhang X, Yin J, et al. Small-airway function variables in spirometry, fractional exhaled nitric oxide, and circulating eosinophils predicted airway hyperresponsiveness in patients with mild asthma. J Asthma Allergy. 2021;14:415–426. doi: 10.2147/JAA.S295345. 33907426 PMC8071078

[45] Hou L, Hao H, Huang G, et al. The value of small airway function parameters and fractional exhaled nitric oxide for predicting positive methacholine challenge test in asthmatics of different ages with FEV1 ≥ 80% predicted. Clin Transl Allergy. 2021;11(1):e12007. doi: 10.1002/clt2.12007. 33900045 PMC8099229

[46] Horváth I, Barnes PJ, Loukides S, et al. A European Respiratory Society technical standard: exhaled biomarkers in lung disease. Eur Respir J. 2017;49(4):1600965. doi: 10.1183/13993003.00965-2016. 28446552

[47] Cattoni I, Guarnieri G, Tosetto A, et al. Mechanisms of decrease in fractional exhaled nitric oxide during acute bronchoconstriction. Chest. 2013;143(5):1269–1276. doi: 10.1378/chest.12-1374. 23370456

