# Supplementary material for: Fractional exhaled nitric oxide at multiple flow rates in chronic cough: association with lung function and airway hyperresponsiveness
Source: Ann Med. 2026 Jun 26;58(1):2681282. doi: 10.1080/07853890.2026.2681282 (PMC13312845; doi:10.1080/07853890.2026.2681282)
Supplement: Supplemental Material [file IANN_A_2681282_SM9430.docx]

**Supporting Information**

*Dan Wang ^1^, Yan Zhang ^1^, Yun Liu ^1*^*

*1 Department of Respiratory and Critical Care Medicine, The Second Affiliated Hospital of Xi'an Jiaotong University, Xi'an 710004, China*

* To whom correspondence should be directed

Email: [yunliusx@163.com](mailto:yunliusx@163.com) (Yun Liu)

Content

[Table S1. Levels of lung function parameters stratified by FeNO_50_ in patients with normal ventilation or obstruction (n=2014). 4](#_Toc215841049)

[Table S2. Comparisons of lung function parameters stratified by FeNO_200_ in patients with normal ventilation or obstruction (n=2014). 4](#_Toc215841050)

[Table S3. Comparisons of lung function parameters stratified by CaNO in patients with normal ventilation or obstruction (n=2014). 5](#_Toc215841051)

[Table S4. Comparisons of lung function parameters stratified by sex in the N+n-SAD group (n = 880). 6](#_Toc215841052)

[Table S5. Comparisons of lung function parameters stratified by sex in the N+ SAD group (n = 399). 7](#_Toc215841053)

[Table S6. Comparisons of lung function parameters stratified by sex in the mild airflow obstruction (n= 542). 8](#_Toc215841054)

[Table S7. Comparisons of lung function parameters stratified by sex in the moderate to extreme airflow obstruction (n=193). 9](#_Toc215841055)

[Table S8. Correlations between FeNO_50_, FeNO_200_, CaNO and lung function parameters in patients with normal ventilation or airway obstruction (n=2014). 10](#_Toc215841056)

[Table S9. Correlations between FeNO_50_, FeNO_200_, CaNO and lung function parameters in female patients with normal ventilation or airway obstruction (n=1086). 11](#_Toc215841057)

[Table S10. Correlations between FeNO_50_, FeNO_200_, CaNO and lung function parameters in male patients with normal ventilation or airway obstruction (n=928). 11](#_Toc215841058)

[Table S11. Correlations between FeNO_50_, FeNO_200_, CaNO and lung function parameters in female patients with N+ n-SAD (n=488). 11](#_Toc215841059)

[Table S12. Correlations between FeNO_50_, FeNO_200_, CaNO and lung function parameters in male patients with N+ n-SAD (n=392). 12](#_Toc215841060)

[Table S13. Correlations between FeNO_50_, FeNO_200_, CaNO and lung function parameters in female patients with N+ SAD (n=243). 12](#_Toc215841061)

[Table S14. Correlations between FeNO_50_, FeNO_200_, CaNO and lung function parameters in male patients with N+ SAD (n=156). 13](#_Toc215841062)

[Table S15. Correlations between FeNO50, FeNO200, CaNO and lung function parameters in female patients with mild obstruction (n=295). 13](#_Toc215841063)

[Table S16. Correlations between FeNO50, FeNO200, CaNO and lung function parameters in male patients with mild obstruction (n=247). 13](#_Toc215841064)

[Table S17. Correlations between FeNO50, FeNO200, CaNO and lung function parameters in female patients with moderate to extreme obstruction (n=60). 14](#_Toc215841065)

[Table S18. Correlations between FeNO50, FeNO200, CaNO and lung function parameters in male patients with moderate to extreme obstruction (n=133). 14](#_Toc215841066)

[Table S19. Comparison of exhaled nitric oxides and baseline lung function parameters in the AHR group and the n-AHR group. 15](#_Toc215841067)

[Table S20. Correlations between exhaled nitric oxides and lung function parameters in the AHR group and the n-AHR group (n=582). 16](#_Toc215841068)

[Table S21. Diagnostic tests of single measurement for the prediction of airway hyperresponsiveness (AHR). 17](#_Toc215841069)

[Table S22. Diagnostic tests of the combined measurements for the prediction of airway hyperresponsiveness (AHR). 18](#_Toc215841070)

[Figure S1. Flow chart of the grouping methods used in this study. 19](#_Toc215841071)

[Figure S2. Levels of exhaled nitric oxides in females and males among patients with different airway function types. 20](#_Toc215841072)

[Figure S3. Levels of exhaled nitric oxides grouped by quartiles of height among patients with different airway function types (n=2014). 21](#_Toc215841073)

[Figure S4. Levels of exhaled nitric oxides grouped by quartiles of weight among patients with different airway function types (n=2014). 22](#_Toc215841074)

[Figure S5. Correlations between exhaled nitric oxides and lung parameters in patients with chronic cough (n=2014). 23](#_Toc215841075)

[Figure S6. Correlations between exhaled nitric oxides and lung parameters in patients with AHR or n-AHR (n=582). 24](#_Toc215841076)

### Table S1. Levels of lung function parameters stratified by FeNO_50_ in patients with normal ventilation or obstruction (n=2014).

|  | FeNO_50_<25 ppb (n=1226) | 25ppb≤FeNO_50_<50 ppb (n=518) | FeNO_50_≥50 ppb (n=270) | *P* value |
| --- | --- | --- | --- | --- |
| FEV_1_(%pred) | 95.61±19.26 | 93.71±19.19 | 89.03±18.04 | 0.003 |
| FEV_1_/FVC  (%pred) | 89.43±13.28 | 88.42±13.41 | 85.86±13.11 | <0.001 |
| FEV_1_/VC(%pred) | 93.68±13.17 | 92.65±13.76 | 88.70±12.72 | <0.001 |
| FEF_50_(%pred) | 69.90±29.25 | 68.15±29.46 | 60.67±27.72 | <0.001 |
| FEF_75_(%pred) | 54.26±26.78 | 52.34±26.39 | 45.74±23.14 | <0.001 |
| FEF_25-75_(%pred) | 63.67±27.00 | 62.20±27.51 | 54.77±25.23 | <0.001 |

Note: Data are presented as means ± SD. All lung function parameters are represented as the percentage of predicted values (%pred).

### Table S2. Comparisons of lung function parameters stratified by FeNO_200_ in patients with normal ventilation or obstruction (n=2014).

|  | FeNO_200_<10 ppb  (n= 765) | FeNO_200_≥10 ppb  (n= 1249) | *P* value |
| --- | --- | --- | --- |
| FEV_1_(%pred) | 96.03±18.26 | 93.14±19.67 | 0.003 |
| FEV_1_/FVC(%pred) | 90.22±12.38 | 87.66±12.83 | <0.001 |
| FEV_1_/VC(%pred) | 94.52±12.39 | 91.66±13.82 | <0.001 |
| FEF_50_(%pred) | 71.35±29.26 | 66.30±29.09 | <0.001 |
| FEF_75_(%pred) | 55.34±26.19 | 50.96±26.34 | <0.001 |
| FEF_25-75_(%pred) | 64.93±26.58 | 60.37±27.20 | <0.001 |

Note: Data are presented as means ± SD. All lung function parameters are represented as the percentage of predicted values (%pred).

### Table S3. Comparisons of lung function parameters stratified by CaNO in patients with normal ventilation or obstruction (n=2014).

|  | CaNO<5 ppb  (n= 804) | CaNO≥5 ppb  (n= 1210) | *P* value |
| --- | --- | --- | --- |
| FEV_1_(%pred) | 95.31±18.60 | 93.52±19.56 | 0.157 |
| FEV_1_/FVC(%pred) | 89.68±12.36 | 87.93±13.93 | 0.010 |
| FEV_1_/VC(%pred) | 93.90±12.32 | 91.98±13.96 | 0.006 |
| FEF_50_(%pred) | 70.00±28.89 | 67.03±29.44 | 0.032 |
| FEF_75_(%pred) | 54.10±25.54 | 51.64±26.86 | 0.009 |
| FEF_25-75_(%pred) | 63.76±26.28 | 61.00±27.50 | 0.017 |

Note: Data are presented as means ± SD. All lung function parameters are represented as the percentage of predicted values (%pred).

### Table S4. Comparisons of lung function parameters stratified by sex in the N+n-SAD group (n = 880).

|  | FeNO_50_<25 ppb (n= 574) | | | 25<FeNO_50_<50 ppb (n= 222) | | | FeNO_50_≥50 ppb (n= 84) | | |
| --- | --- | --- | --- | --- | --- | --- | --- | --- | --- |
|  | Female (n= 354) | Male (n= 220) | *P* value | Female (n= 104) | Male (n= 118) | *P* value | Female (n= 40) | Male (n= 44) | *P* value |
| FEV_1_(%pred) | 107.96±13.17 | 105.90±10.95 | 0.002 | 107.80±13.02 | 105.90±10.00 | 0.007 | 105.05±11.12 | 104.62±10.25 | 0.837 |
| FEV_1_/FVC(%pred) | 99.00±6.32 | 97.86±6.52 | 0.165 | 99.45±5.59 | 96.74±7.46 | 0.302 | 100.719±6.86 | 96.91±4.49 | 0.103 |
| FEV_1_/VC(%pred) | 102.78±4.82 | 101.77±6.09 | 0.007 | 102.46±5.86 | 102.46±5.86 | 0.152 | 101.82±5.04 | 101.52±4.49 | 0.688 |
| FEF_50_(%pred) | 93.41±18.52 | 95.48±20.03 | 0.197 | 92.20±16.43 | 96.91±23.00 | 0.035 | 91.43±15.88 | 94.63±15.63 | 0.823 |
| FEF_75_(%pred) | 72.62±20.84 | 78.06±24.92 | 0.037 | 74.19±18.28 | 76.19±25.60 | 0.013 | 71.86±73.71 | 73.71±17.14 | 0.864 |
| FEF_25-75_(%pred) | 85.28±16.2S0 | 88.61±18.77 | 0.030 | 85.85±13.60 | 89.59±22.09 | 0.006 | 83.98±14.73 | 86.56±13.03 | 0.359 |

Note: Data are presented as means ± SD. All lung function parameters are represented as the percentage of predicted values (%pred). All *P* values are for between-sex comparisons within each FeNO_50_ subgroup.

### Table S5. Comparisons of lung function parameters stratified by sex in the N+ SAD group (n = 399).

|  | FeNO_50_<25 ppb (n= 244) | | | 25<FeNO_50_<50 ppb (n= 118) | | | FeNO_50_≥50 ppb (n= 37) | | |
| --- | --- | --- | --- | --- | --- | --- | --- | --- | --- |
|  | Female (n= 155) | Male (n= 89) | *P* value | Female (n= 69) | Male (n= 49) | *P* value | Female (n=19) | Male (18) | *P* value |
| FEV_1_(%pred) | 96.73±12.31 | 93.21±9.00 | 0.028 | 94.39±10.45 | 92.31±9.61 | 0.113 | 96.18±8.63 | 90.19±11.54 | *0.472* |
| FEV_1_/FVC(%pred) | 90.70±4.87 | 88.63±3.43 | 0.355 | 90.90±2.82 | 89.22±3.37 | 0.109 | 91.61±3.25 | 89.58±4.96 | 0.186 |
| FEV_1_/VC(%pred) | 96.15±2.67 | 95.15±2.59 | 0.474 | 96.37±2.67 | 95.79±2.66 | 0.860 | 95.64±2.02 | 95.09±2.90 | 0.303 |
| FEF_50_(%pred) | 62.95±10.54 | 63.83±9.78 | 0.395 | 60.17±10.88 | 65.35±9.54 | 0.519 | 67.26±8.59 | 64.60±13.87 | 0.279 |
| FEF_75_(%pred) | 42.11±8.43 | 44.02±9.70 | 0.342 | 43.00±8.27 | 42.45±9.66 | 0.324 | 45.60±13.87 | 47.23±6.92 | 0.846 |
| FEF_25-75_(%pred) | 54.32±8.45 | 55.60±7.06 | 0.073 | 53.55±6.09 | 55.58±7.13 | 0.528 | 58.71±5.42 | 55.98±8.70 | 0.174 |

Note: Data are presented as means ± SD. All lung function parameters are represented as the percentage of predicted values (%pred). All P values are for between-sex comparisons within each FeNO_50_ subgroup.

### Table S6. Comparisons of lung function parameters stratified by sex in the mild airflow obstruction (n= 542).

|  | FeNO_50_<25 ppb (n= 300) | | | 25<FeNO_50_<50 ppb (n= 128) | | | FeNO_50_≥50 ppb (n= 114) | | |
| --- | --- | --- | --- | --- | --- | --- | --- | --- | --- |
|  | Female (n= 157) | Male (n= 143) | *P* value | Female (n= 68) | Male (n= 60) | *P* value | Female (n= 62) | Male (n= 52) | *P* value |
| FEV_1_(%pred) | 89.97±12.27 | 89.37±11.29 | 0.418 | 86.83±13.23 | 88.50±10.56 | 0.338 | 87.24±12.50 | 84.94±10.33 | 0.241 |
| FEV_1_/FVC(%pred) | 82.46±5.64 | 80.08±4.96 | 0.258 | 80.91±6.16 | 79.61±6.83 | 0.119 | 81.19±6.50 | 79.07±7.83 | 0.456 |
| FEV_1_/VC(%pred) | 86.07±4.96 | 84.93±6.31 | 0.167 | 84.18±6.55 | 83.42±7.54 | 0.440 | 83.89±6.23 | 83.13±7.57 | 0.417 |
| FEF_50_(%pred) | 45.52±12.08 | 49.50±11.94 | 0.738 | 43.52±11.98 | 48.08±11.73 | 0.826 | 45.68±13.67 | 47.89±13.02 | 0.693 |
| FEF_75_(%pred) | 33.94±10.07 | 38.25±12.00 | 0.170 | 32.54±11.24 | 35.38±11.04 | 0.939 | 31.82±9.96 | 35.76±9.77 | 0.790 |
| FEF_25-75_(%pred) | 40.94±10.41 | 45.22±11.52 | 0.172 | 39.64±12.67 | 43.54±10.30 | 0.397 | 40.30±12.12 | 43.15±10.99 | 0.621 |

Note: Data are presented as means ± SD. All lung function parameters are represented as the percentage of predicted values (%pred). All P values are for between-sex comparisons within each FeNO50 subgroup.

### Table S7. Comparisons of lung function parameters stratified by sex in the moderate to extreme airflow obstruction (n=193).

|  | FeNO_50_<25 ppb (n= 112) | | *P* value | 25<FeNO_50_<50 ppb (n= 49) |  | *P* value | FeNO_50_≥50 ppb (n= 32) |  | *P* value |
| --- | --- | --- | --- | --- | --- | --- | --- | --- | --- |
|  | Female (n= 36) | Male (n= 76) |  | Female (n= 13) | Male (n= 36) |  | Female (n= 11) | Male (n= 21) |  |
| FEV_1_(%pred) | 57.07±11.15 | 51.18±14.03 | 0.098 | 56.42±15.57 | 50.84±13.40 | 0.761 | 60.25±5.36 | 55.82±9.55 | 0.076 |
| FEV_1_/FVC(%pred) | 65.29±10.37 | 57.89±13.20 | 0.025 | 66.98±16.77 | 57.87±12.19 | 0.265 | 66.81±7.68 | 63.43±11.65 | 0.169 |
| FEV_1_/VC(%pred) | 67.55±11.15 | 61.93±14.16 | 0.023 | 67.42±16.48 | 61.38±14.03 | 0.630 | 70.06±6.49 | 66.99±10.93 | 0.103 |
| FEF_50_(%pred) | 19.72±14.16 | 18.94±9.47 | 0.030 | 22.28±10.58 | 19.56±12.43 | 0.693 | 21.79±7.18 | 22.14±9.28 | 0.327 |
| FEF_75_(%pred) | 16.15±5.72 | 18.48±7.56 | 0.078 | 17.12±6.10 | 16.68±6.13 | 0.916 | 18.14±6.10 | 18.60±5.35 | 0.469 |
| FEF_25-75_(%pred) | 18.20±6.55 | 18.43±8.33 | 0.031 | 19.77±9.17 | 17.69±8.13 | 0.590 | 25.40±15.56 | 20.88±7.46 | 0.058 |

Note: Data are presented as means ± SD. All lung function parameters are represented as the percentage of predicted values (%pred). All P values are for between-sex comparisons within each FeNO_50_ subgroup.

### Table S8. Correlations between FeNO_50_, FeNO_200_, CaNO and lung function parameters in patients with normal ventilation or airway obstruction (n=2014).

|  | FeNO_50_ | | FeNO_200_ | | CaNO | |
| --- | --- | --- | --- | --- | --- | --- |
|  | *r* | *P* | *r* | *P* | *r* | *P* |
| FEV_1_(%pred) | -0.097 | <0.001 | -0.097 | <0.001 | -0.024 | 0.255 |
| FEV_1_/FVC(%pred) | -0.124 | <0.001 | -0.127 | <0.001 | -0.055 | 0.009 |
| FEV_1_/VC(%pred) | -0.137 | <0.001 | -0.144 | <0.001 | -0.055 | 0.009 |
| FEF_50_(%pred) | -0.096 | <0.001 | -0.100 | <0.001 | -0.035 | 0.102 |
| FEF_75_(%pred) | -0.106 | <0.001 | -0.117 | <0.001 | -0.062 | 0.004 |
| FEF_25-75_(%pred) | -0.101 | <0.001 | -0.108 | <0.001 | -0.044 | 0.039 |

Note: No parametric Spearman’s rank correlations are used, two-tailed *P* value and *r* for each pair of exhaled nitric oxides and lung function parameters are presented.

### Table S9. Correlations between FeNO_50_, FeNO_200_, CaNO and lung function parameters in female patients with normal ventilation or airway obstruction (n=1086).

|  | FeNO_50_ | | FeNO_200_ | | CaNO | |
| --- | --- | --- | --- | --- | --- | --- |
|  | *r* | *P* | *r* | *P* | *r* | *P* |
| FEV_1_(%pred) | -0.111 | <0.001 | -0.109 | <0.001 | -0.020 | 0.503 |
| FEV_1_/FVC(%pred) | -0.135 | <0.001 | -0.131 | <0.001 | -0.049 | 0.109 |
| FEV_1_/VC(%pred) | -0.162 | <0.001 | -0.164 | <0.001 | -0.058 | 0.055 |
| FEF_50_(%pred) | -0.132 | <0.001 | -0.140 | <0.001 | -0.063 | 0.038 |
| FEF_75_(%pred) | -0.121 | <0.001 | -0.135 | <0.001 | -0.065 | 0.033 |
| FEF_25-75_(%pred) | -0.127 | <0.001 | -0.140 | <0.001 | -0.065 | 0.031 |

Note: No parametric Spearman’s rank correlations are used, two-tailed *P* value and *r* for each pair of exhaled nitric oxides and lung function parameters are presented.

### Table S10. Correlations between FeNO_50_, FeNO_200_, CaNO and lung function parameters in male patients with normal ventilation or airway obstruction (n=928).

|  | FeNO_50_ | | FeNO_200_ | | CaNO | |
| --- | --- | --- | --- | --- | --- | --- |
|  | *r* | *P* | *r* | *P* | *r* | *P* |
| FEV_1_(%pred) | -0.091 | 0.006 | -0.071 | 0.031 | 0.018 | 0.587 |
| FEV_1_/FVC(%pred) | -0.066 | 0.045 | -0.081 | 0.014 | -0.025 | 0.448 |
| FEV_1_/VC(%pred) | -0.078 | 0.017 | -0.098 | 0.003 | -0.029 | 0.372 |
| FEF_50_(%pred) | -0.051 | 0.123 | -0.062 | 0.059 | -0.009 | 0.789 |
| FEF_75_(%pred) | -0.095 | 0.004 | -0.102 | 0.002 | -0.047 | 0.153 |
| FEF_25-75_(%pred) | -0.073 | 0.026 | -0.083 | 0.012 | -0.022 | 0.500 |

Note: No parametric Spearman’s rank correlations are used, two-tailed *P* value and r for each pair of exhaled nitric oxides and lung function parameters are presented.

### Table S11. Correlations between FeNO_50_, FeNO_200_, CaNO and lung function parameters in female patients with N+ n-SAD (n=488).

|  | FeNO_50_ | | FeNO_200_ | | CaNO | |
| --- | --- | --- | --- | --- | --- | --- |
|  | *r* | *P* | *r* | *P* | *r* | *P* |
| FEV_1_(%pred) | -0.02 | 0.681 | -0.018 | 0.708 | 0.032 | 0.551 |
| FEV_1_/FVC(%pred) | 0.029 | 0.541 | 0.032 | 0.511 | -0.018 | 0.713 |
| FEV_1_/VC(%pred) | -0.037 | 0.438 | -0.073 | 0.131 | -0.037 | 0.441 |
| FEF_50_(%pred) | 0.006 | 0.906 | -0.030 | 0.538 | -0.016 | 0.741 |
| FEF_75_(%pred) | 0.045 | 0.348 | 0.007 | 0.882 | -0.010 | 0.843 |
| FEF_25-75_(%pred) | 0.035 | 0.466 | -0.003 | 0.946 | 0.003 | 0.948 |

Note: No parametric Spearman’s rank correlations are used, two-tailed *P* value and r for each pair of exhaled nitric oxides and lung function parameters are presented.

### Table S12. Correlations between FeNO_50_, FeNO_200_, CaNO and lung function parameters in male patients with N+ n-SAD (n=392).

|  | FeNO_50_ | | FeNO_200_ | | CaNO | |
| --- | --- | --- | --- | --- | --- | --- |
|  | *r* | *P* | *r* | *P* | *r* | *P* |
| FEV_1_(%pred) | -0.016 | 0.776 | 0.057 | 0.297 | 0.132 | 0.016 |
| FEV_1_/FVC(%pred) | -0.067 | 0.221 | -0.061 | 0.265 | -0.039 | 0.483 |
| FEV_1_/VC(%pred) | -0.023 | 0.682 | -0.042 | 0.448 | -0.017 | 0.751 |
| FEF_50_(%pred) | -0.010 | 0.858 | -0.001 | 0.984 | 0.029 | 0.597 |
| FEF_75_(%pred) | -0.071 | 0.194 | -0.059 | 0.282 | -0.032 | 0.556 |
| FEF_25-75_(%pred) | -0.021 | 0.698 | -0.002 | 0.969 | 0.043 | 0.439 |

Note: No parametric Spearman’s rank correlations are used, two-tailed *P* value and r for each pair of exhaled nitric oxides and lung function parameters are presented.

### Table S13. Correlations between FeNO_50_, FeNO_200_, CaNO and lung function parameters in female patients with N+ SAD (n=243).

|  | FeNO_50_ | | FeNO_200_ | | CaNO | |
| --- | --- | --- | --- | --- | --- | --- |
|  | *r* | *P* | *r* | *P* | *r* | *P* |
| FEV_1_(%pred) | -0.016 | 0.819 | -0.029 | 0.669 | 0.057 | 0.403 |
| FEV_1_/FVC(%pred) | -0.03 | 0.661 | -0.003 | 0.960 | 0.001 | 0.991 |
| FEV_1_/VC(%pred) | 0.013 | 0.853 | 0.022 | 0.753 | 0.045 | 0.513 |
| FEF_50_(%pred) | -0.044 | 0.517 | -0.011 | 0.878 | -0.044 | 0.524 |
| FEF_75_(%pred) | 0.050 | 0.468 | 0.033 | 0.632 | 0.013 | 0.848 |
| FEF_25-75_(%pred) | -0.012 | 0.859 | -0.010 | 0.880 | -0.051 | 0.456 |

Note: No parametric Spearman’s rank correlations are used, two-tailed P value and r for each pair of exhaled nitric oxides and lung function parameters are presented.

### Table S14. Correlations between FeNO_50_, FeNO_200_, CaNO and lung function parameters in male patients with N+ SAD (n=156).

|  | FeNO_50_ | | FeNO_200_ | | CaNO | |
| --- | --- | --- | --- | --- | --- | --- |
|  | *r* | *P* | *r* | *P* | *r* | *P* |
| FEV_1_(%pred) | -0.118 | 0.148 | -0.092 | 0.260 | -0.054 | 0.509 |
| FEV_1_/FVC(%pred) | 0.124 | 0.129 | 0.058 | 0.477 | -0.032 | 0.697 |
| FEV_1_/VC(%pred) | 0.041 | 0.615 | -0.044 | 0.595 | -0.097 | 0.235 |
| FEF_50_(%pred) | 0.081 | 0.322 | 0.051 | 0.618 | -0.024 | 0.774 |
| FEF_75_(%pred) | -0.055 | 0.502 | -0.090 | 0.269 | -0.128 | 0.118 |
| FEF_25-75_(%pred) | 0.019 | 0.817 | -0.031 | 0.706 | -0.091 | 0.264 |

Note: No parametric Spearman’s rank correlations are used, two-tailed P value and r for each pair of exhaled nitric oxides and lung function parameters are presented.

### Table S15. Correlations between FeNO50, FeNO200, CaNO and lung function parameters in female patients with mild obstruction (n=295).

|  | FeNO_50_ | | FeNO_200_ | | CaNO | |
| --- | --- | --- | --- | --- | --- | --- |
|  | *r* | *P* | *r* | *P* | *r* | *P* |
| FEV_1_(%pred) | -0.080 | 0.198 | -0.095 | 0.125 | 0.010 | 0.869 |
| FEV_1_/FVC(%pred) | -0.068 | 0.276 | -0.083 | 0.178 | 0.030 | 0.630 |
| FEV_1_/VC(%pred) | -0.155 | 0.012 | -0.148 | 0.016 | 0.011 | 0.861 |
| FEF_50_(%pred) | -0.010 | 0.868 | -0.052 | 0.406 | -0.002 | 0.972 |
| FEF_75_(%pred) | -0.074 | 0.232 | -0.103 | 0.095 | -0.038 | 0.545 |
| FEF_25-75_(%pred) | -0.022 | 0.725 | -0.067 | 0.292 | -0.019 | 0.761 |

Note: No parametric Spearman’s rank correlations are used, two-tailed P value and r for each pair of exhaled nitric oxides and lung function parameters are presented.

### Table S16. Correlations between FeNO50, FeNO200, CaNO and lung function parameters in male patients with mild obstruction (n=247).

|  | FeNO_50_ | | FeNO_200_ | | CaNO | |
| --- | --- | --- | --- | --- | --- | --- |
|  | *r* | *P* | *r* | *P* | *r* | *P* |
| FEV_1_(%pred) | -0.143 | 0.029 | -0.093 | 0.156 | 0.066 | 0.316 |
| FEV_1_/FVC(%pred) | -0.077 | 0.241 | -0.021 | 0.746 | 0.061 | 0.355 |
| FEV_1_/VC(%pred) | -0.148 | 0.024 | -0.102 | 0.120 | -0.003 | 0.962 |
| FEF_50_(%pred) | -0.091 | 0.166 | -0.019 | 0.767 | 0.080 | 0.222 |
| FEF_75_(%pred) | -0.138 | 0.036 | -0.095 | 0.150 | -0.034 | 0.608 |
| FEF_25-75_(%pred) | -0.108 | 0.100 | -0.048 | 0.463 | 0.046 | 0.485 |

Note: No parametric Spearman’s rank correlations are used, two-tailed P value and r for each pair of exhaled nitric oxides and lung function parameters are presented.

### Table S17. Correlations between FeNO50, FeNO200, CaNO and lung function parameters in female patients with moderate to extreme obstruction (n=60).

|  | FeNO_50_ | | FeNO_200_ | | CaNO | |
| --- | --- | --- | --- | --- | --- | --- |
|  | *r* | *P* | *r* | *P* | *r* | *P* |
| FEV_1_(%pred) | -0.061 | 0.663 | -0.039 | 0.783 | 0.001 | 0.996 |
| FEV_1_/FVC(%pred) | 0.010 | 0.942 | 0..043 | 0.758 | 0.010 | 0.942 |
| FEV_1_/VC(%pred) | -0.025 | 0.858 | -0.039 | 0.780 | -0.091 | 0.517 |
| FEF_50_(%pred) | -0.019 | 0.894 | 0.015 | 0.918 | -0.037 | 0.795 |
| FEF_75_(%pred) | -0.056 | 0.688 | -0.088 | 0.532 | -0.150 | 0.284 |
| FEF_25-75_(%pred) | -0.040 | 0.776 | -0.009 | 0.951 | -0.044 | 0.756 |

Note: No parametric Spearman’s rank correlations are used, two-tailed P value and r for each pair of exhaled nitric oxides and lung function parameters are presented.

### Table S18. Correlations between FeNO50, FeNO200, CaNO and lung function parameters in male patients with moderate to extreme obstruction (n=133).

|  | FeNO_50_ | | FeNO_200_ | | CaNO | |
| --- | --- | --- | --- | --- | --- | --- |
|  | *r* | *P* | *r* | *P* | *r* | *P* |
| FEV_1_(%pred) | 0.051 | 0.561 | 0.023 | 0.793 | -0.025 | 0.781 |
| FEV_1_/FVC(%pred) | 0.093 | 0.292 | 0.039 | 0.660 | -0.080 | 0.366 |
| FEV_1_/VC(%pred) | 0.084 | 0.339 | 0.037 | 0.671 | -0.064 | 0.467 |
| FEF_50_(%pred) | 0.061 | 0.489 | 0.032 | 0.718 | -0.047 | 0.593 |
| FEF_75_(%pred) | -0.045 | 0.606 | -0.040 | 0.650 | -0.052 | 0.554 |
| FEF_25-75_(%pred) | 0.057 | 0.521 | 0.026 | 0.772 | -0.059 | 0.502 |

Note: No parametric Spearman’s rank correlations are used, two-tailed P value and r for each pair of exhaled nitric oxides and lung function parameters are presented.

### Table S19. Comparison of exhaled nitric oxides and baseline lung function parameters in the AHR group and the n-AHR group.

|  | AHR (n= 156) | n-AHR (n= 426) | *P* value |
| --- | --- | --- | --- |
| FeNO_50_ | 55.14±40.04 | 21.93±16.96 | <0.001 |
| FeNO_200_ | 21.35±14.17 | 11.71±8.53 | <0.001 |
| CaNO | 6.47±5.90 | 7.33±7.62 | 0.125 |
| FEV_1_(%pred) | 91.54±12.98 | 100.93±12.15 | 0.157 |
| FEV_1_/FVC(%pred) | 86.97±9.05 | 95.77±7.83 | 0.010 |
| FEV_1_/VC(%pred) | 89.86±77.60 | 98.64±7.28 | 0.006 |
| FEF_50_(%pred) | 58.37±18.89 | 81.79±22.62 | 0.032 |
| FEF_75_(%pred) | 45.69±18.28 | 65.81±25.81 | 0.009 |
| FEF_25-75_(%pred) | 53.63±17.91 | 75.85±22.48 | 0.017 |

Note: Data are presented as means ± SD.

Abbreviations: AHR, airway hyperresponsiveness; N-AHR, normal airway responsiveness.

### Table S20. Correlations between exhaled nitric oxides and lung function parameters in the AHR group and the n-AHR group (n=582).

|  | FeNO_50_ | | FeNO_200_ | | CaNO | |
| --- | --- | --- | --- | --- | --- | --- |
|  | *r* | *P* | *r* | *P* | *r* | *P* |
| FEV_1_(%pred) | -0.112 | 0.007 | -0.094 | 0.023 | 0.015 | 0.711 |
| FEV_1_/FVC(%pred) | -0.123 | 0.003 | -0.109 | 0.008 | 0.019 | 0.647 |
| FEV_1_/VC(%pred) | -0.131 | 0.002 | -0.119 | 0.004 | 0.021 | 0.621 |
| FEF_50_(%pred) | -0.102 | 0.014 | -0.088 | 0.034 | 0.030 | 0.471 |
| FEF_75_(%pred) | -0.115 | 0.005 | -0.099 | 0.017 | 0.025 | 0.545 |
| FEF_25-75_(%pred) | -0.119 | 0.004 | -0.105 | 0.011 | 0.027 | 0.522 |

Note: No parametric Spearman’ rank correlations are used, two-tailed p value and r for each pair of exhaled nitric oxides and lung function parameters are presented.

### Table S21. Diagnostic tests of single measurement for the prediction of airway hyperresponsiveness (AHR).

| Variables | AUC (95%CI) | Cutoff values | Sensitivity (%) | Specificity (%) | Youden index | *P* value |
| --- | --- | --- | --- | --- | --- | --- |
| FeNO_50_ | 0.775(0.727,0.823) | 31.5 | 62.8 | 84.5 | 1.473 | <0.001 |
| FeNO_200_ | 0.721(0.670,0.771) | 14.5 | 60.9 | 77.2 | 1.381 | <0.001 |
| CaNO | 0.459(0.405,0.513) | - | - | - | - | 0.125 |
| FEV_1_(%pred) | 0.708(0.658,0.785) | 91.55 | 56.4 | 78.2 | 1.346 | <0.001 |
| FEV_1_/FVC(%pred) | 0.775(0.730,0.819) | 88.05 | 59.6 | 82.6 | 1.422 | <0.001 |
| FEV_1_/VC(%pred) | 0.782(0.738,0.826) | 93.15 | 64.1 | 89.3 | 1.434 | <0.001 |
| FEF_50_(%pred) | 0.785(0.744,0.827) | 64.05 | 64.7 | 76.8 | 1.415 | <0.001 |
| FEF_75_(%pred) | 0.745(0.700,0.790) | 40.95 | 50.0 | 86.7 | 1.357 | <0.001 |
| FEF_25-75_(%pred) | 0.780(0.738,0.822) | 53.25 | 55.1 | 85.7 | 1.408 | <0.001 |

Note: Cutoff points of FENO_50_ and FENO_200_ are in ppb. The cutoff points were selected by maximizing the sum of sensitivity and speciﬁcity.

Abbreviation: AUC, Area under the curve;

### Table S22. Diagnostic tests of the combined measurements for the prediction of airway hyperresponsiveness (AHR).

| Variables | AUC (95%CI) | Sensitivity (%) | Specificity (%) | Youden index |  |  | *P* value |
| --- | --- | --- | --- | --- | --- | --- | --- |
| FeNO_50_+FeNO_200_ | 0.805(0.762,0.849) | 55.1 | 85.1 | 1.502 |  |  | <0.001 |
| FeNO_50_+FEV_1_(%pred) | 0.845(0.806,0.884) | 74.4 | 84.3 | 1.587 |  |  | <0.001 |
| FeNO_50+_FEV_1_/FVC(%pred) | 0.870(0.834,0.905) | 70.5 | 90.6 | 1.611 |  |  | <0.001 |
| FeNO_50+_FEV_1_/VC(%pred) | 0.872(0.837,0.908) | 71.8 | 92.3 | 1.641 |  |  | <0.001 |
| FeNO_50+_FEF_50_(%pred) | 0.895(0.865,0.925) | 85.9 | 80.8 | 1.667 |  |  | <0.001 |
| FeNO_50+_FEF_75_(%pred) | 0.856(0.821,0.892) | 67.9 | 88.5 | 1.564 |  |  | <0.001 |
| FeNO_50+_FEF_25-75_(%pred) | 0.886(0.855,0.917) | 89.1 | 73.0 | 1.621 |  |  | <0.001 |
| FeNO_200_+FEV_1_(%pred) | 0.800(0.758,0.843) | 66.0 | 83.6 | 1.496 |  |  | <0.001 |
| FeNO_200+_FEV_1_/FVC(%pred) | 0.834(0.796,0.872) | 75.0 | 78.4 | 1.534 |  |  | <0.001 |
| FeNO_200+_FEV_1_/VC(%pred) | 0.839(0.801,0.877) | 73.7 | 81.0 | 1.547 |  |  | <0.001 |
| FeNO_200+_FEF_50_(%pred) | 0.857(0.823,0.890) | 84.0 | 73.5 | 1.575 |  |  | <0.001 |
| FeNO_200+_FEF_75_(%pred) | 0.820(0.782,0.859) | 71.8 | 78.2 | 1.500 |  |  | <0.001 |
| FeNO_200+_FEF_25-75_(%pred) | 0.848(0.814,0.882) | 83.3 | 71.8 | 1.551 |  |  | <0.001 |

Abbreviation: AUC, Area under the curve;


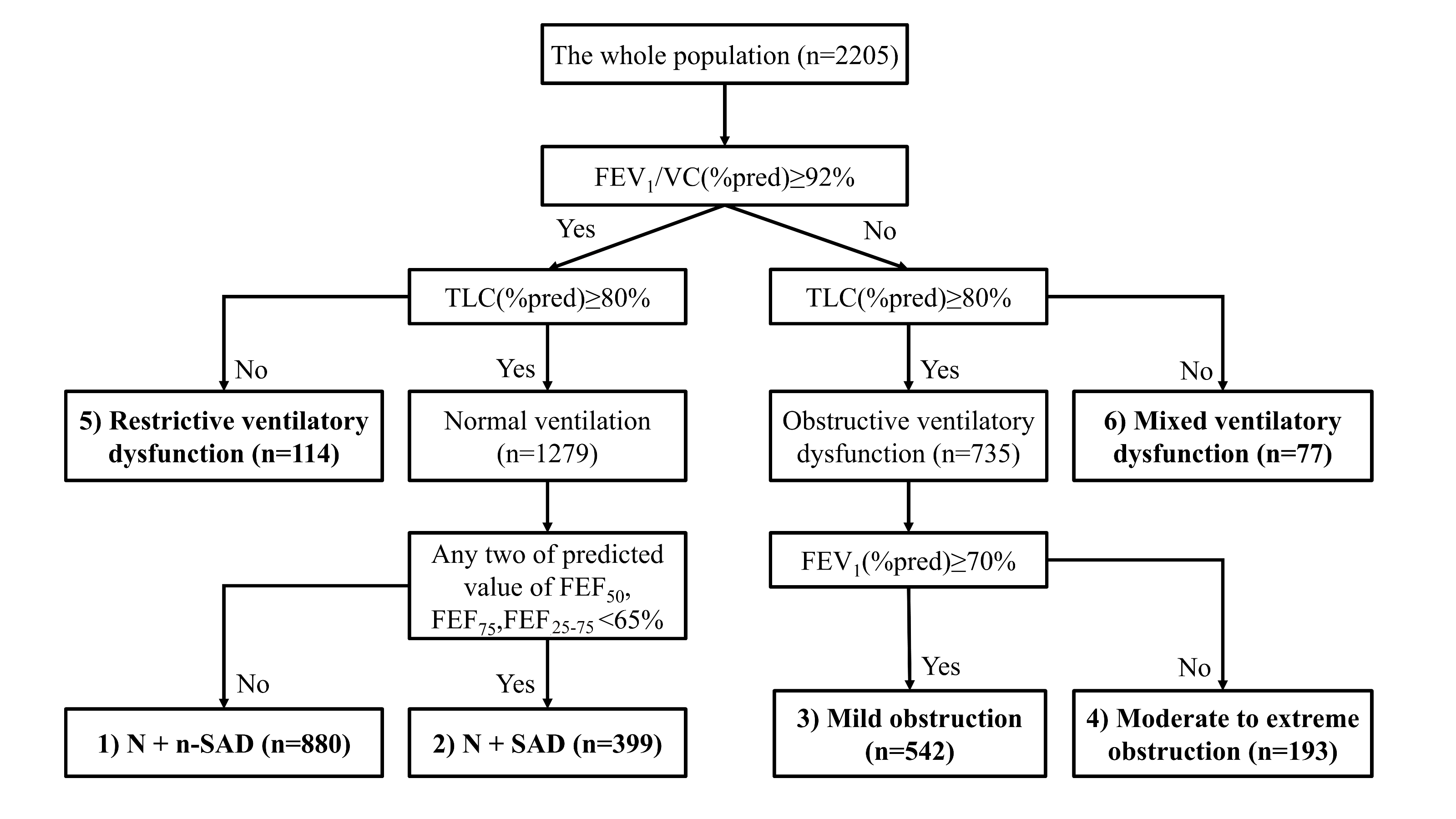


### Figure S1. Flow chart of the grouping methods used in this study.

### Figure S2. Levels of exhaled nitric oxides in females and males among patients with different airway function types.

Note: Independent-samples Kruskal-Wallis tests and pairwise comparisons among multiple groups are used. Mean values in each subgroup are annotated within bars. ***, *P*<0.001; **, *P*<0.01; *, *P*<0.05; ns, no significance.


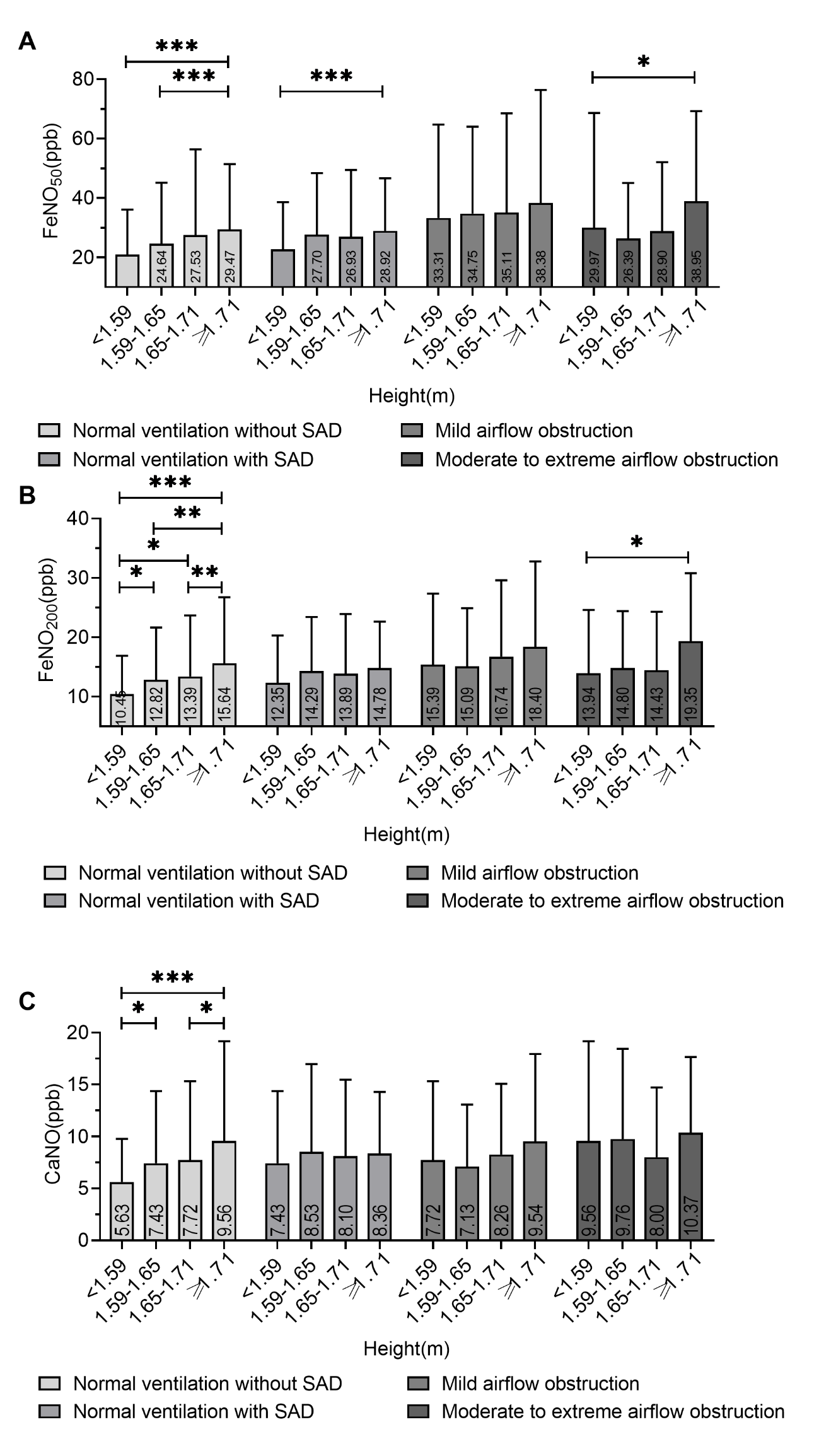


### Figure S3. Levels of exhaled nitric oxides grouped by quartiles of height among patients with different airway function types (n=2014).

Note: Independent-samples Kruskal-Wallis tests and pairwise comparisons among multiple groups are used. ***, *P*<0.001; **, *P*<0.01; *, *P*<0.05.


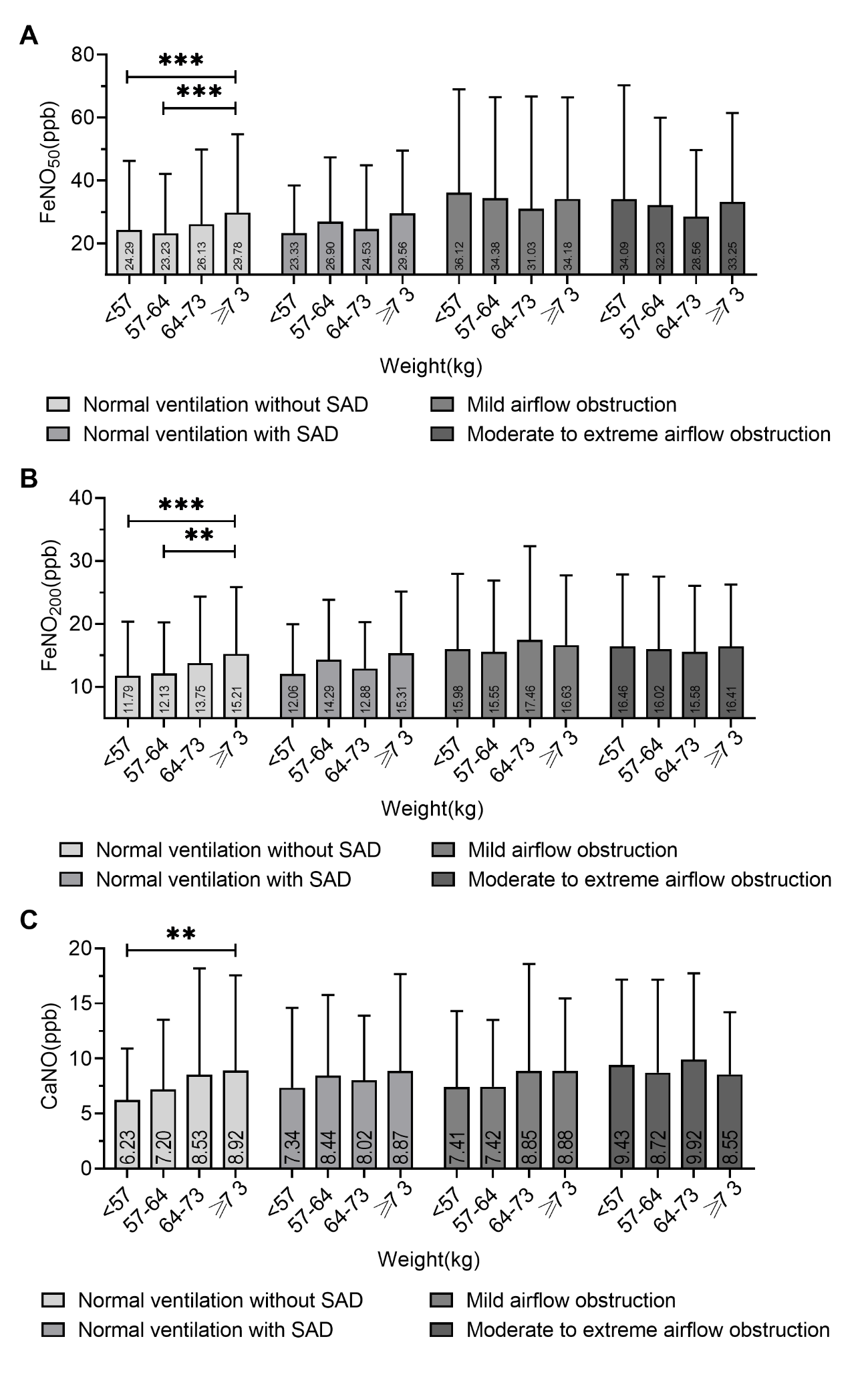


### Figure S4. Levels of exhaled nitric oxides grouped by quartiles of weight among patients with different airway function types (n=2014).

Note: Independent-samples Kruskal-Wallis tests and pairwise comparisons among multiple groups are used. ***, *P*<0.001; **, *P*<0.01; *, *P*<0.05.


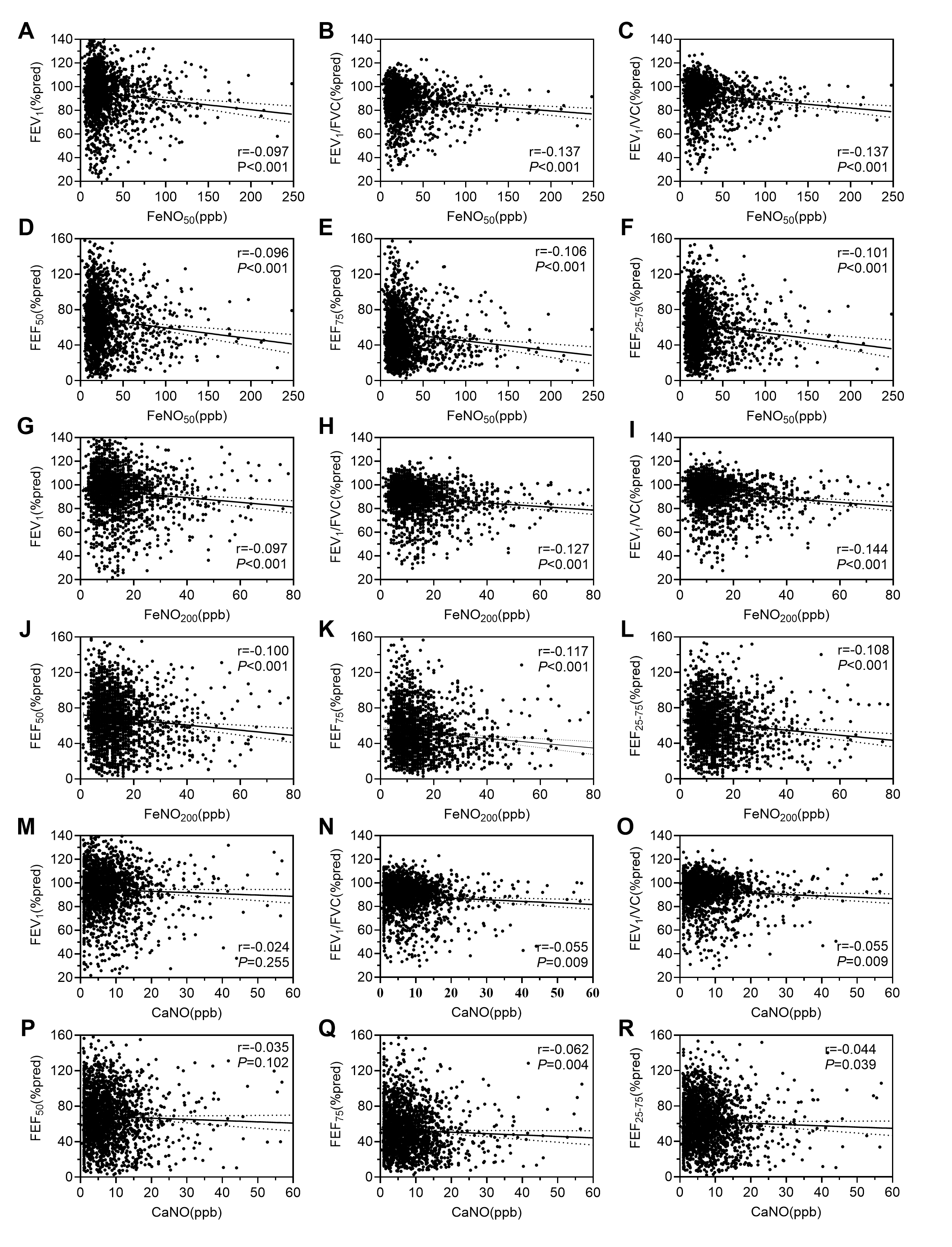


### Figure S5. Correlations between exhaled nitric oxides and lung parameters in patients with chronic cough (n=2014).

Note: Straight line is fitted as the best-fit line with linear regression, and 95% confidence bands are represented with dotted lines in each figure. Spearman r (95% confidence interval) and two-tailed P value are analyzed by nonparametric Spearman’s rank correlation.


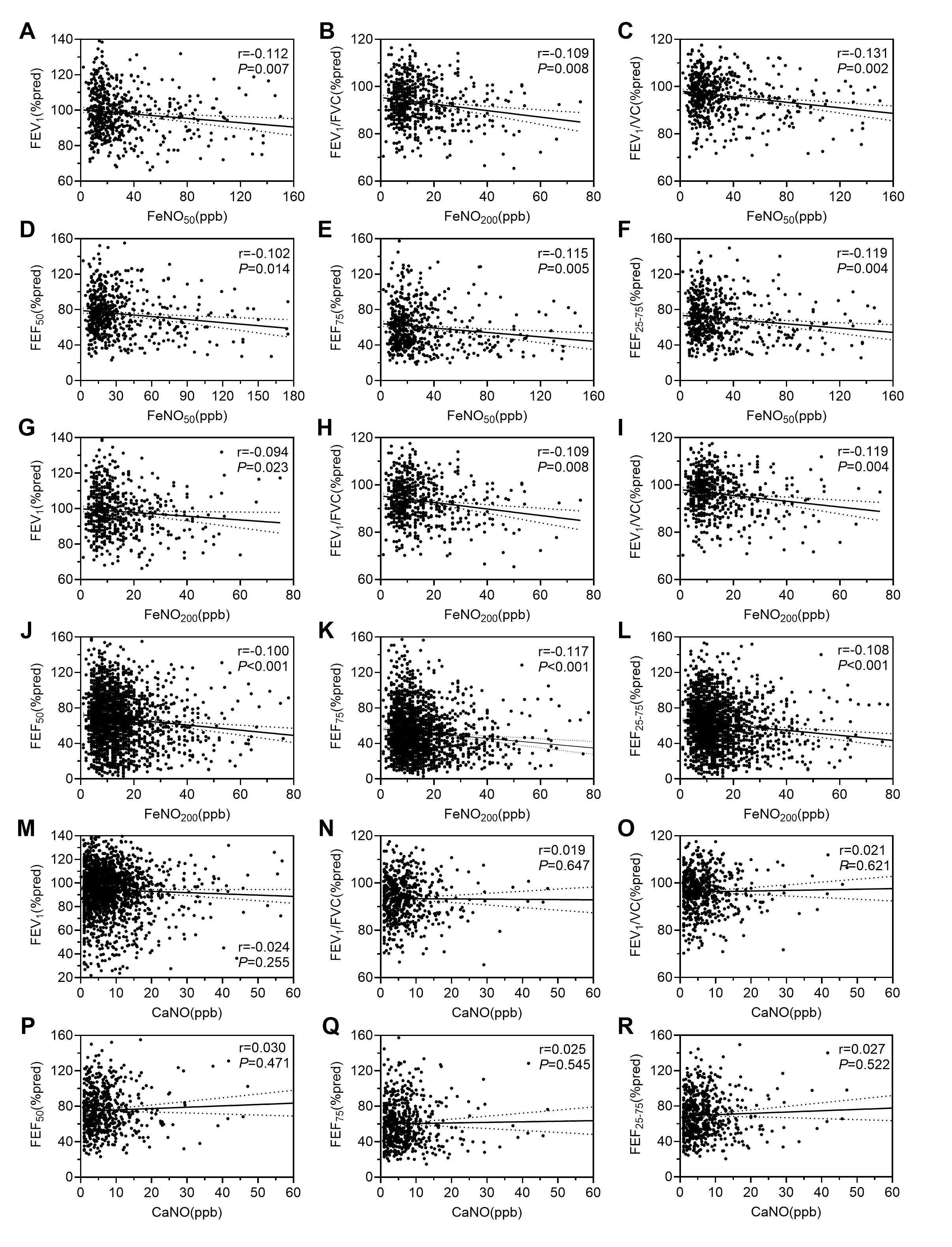


### Figure S6. Correlations between exhaled nitric oxides and lung parameters in patients with AHR or n-AHR (n=582).

Note: Straight line is fitted as the best-fit line with linear regression, and 95% confidence bands are represented with dotted lines in each figure. Spearman r (95% confidence interval) and two-tailed *P* value are analyzed by nonparametric Spearman’s rank correlation.
